# Disparity between Inter-Patient Molecular Heterogeneity and Repertoires of Target Drugs Used for Different Types of Cancer in Clinical Oncology

**DOI:** 10.3390/ijms21051580

**Published:** 2020-02-26

**Authors:** Marianna A. Zolotovskaia, Maxim I. Sorokin, Ivan V. Petrov, Elena V. Poddubskaya, Alexey A. Moiseev, Marina I. Sekacheva, Nicolas M. Borisov, Victor S. Tkachev, Andrew V. Garazha, Andrey D. Kaprin, Peter V. Shegay, Alf Giese, Ella Kim, Sergey A. Roumiantsev, Anton A. Buzdin

**Affiliations:** 1 Oncobox ltd., Moscow, 121205, Russia; petrov@oncobox.com (I.V.P.); buzdin@oncobox.com (A.A.B.); 2 Department of Oncology, Hematology and Radiotherapy of Pediatric Faculty, Pirogov Russian National Research Medical University, Moscow, 117997, Russia; roumiantsev_sa@rsmu.ru; 3 Moscow Institute of Physics and Technology, Dolgoprudny, Moscow Region 141701, Russia; borisov@oncobox.com; 4 I.M. Sechenov First Moscow State Medical University, Moscow, 119991, Russiapodd-elena@yandex.ru (E.V.P.); moiseeev.aa@yusupovs.ru (A.A.M.);; 5 Omicsway Corp., Walnut, CA, 91789, USA; tkachev@oncobox.com (V.S.T.); garazha@oncobox.com (A.V.G.); 6 Shemyakin-Ovchinnikov Institute of Bioorganic Chemistry, Moscow, 117997, Russia; 7 National Medical Research Radiological Centre of the Ministry of Health of the Russian Federation, Moscow 125284, Russia; kaprin@mail.ru; 8 Center for Innovative Radiological and Regenerative Technologies of the Ministry of Health of the Russian Federation, Obninsk 249030, Russia; dr.shegai@mail.ru; 9 Orthocentrum Hamburg, Hamburg, Germany; alf.giese1@gmail.com or; 10 Johannes Gutenberg University Mainz, Mainz, Germany; ella.kim@gmx.de

**Keywords:** clinical oncology, targeted therapeutics, cancer drugs, chemotherapy, molecular diagnostics, mutations, genome, transcriptome, tumor heterogeneity, personalized medicine

## Abstract

Inter-patient molecular heterogeneity is the major declared driver of an expanding variety of anticancer drugs and personalizing their prescriptions. Here, we compared interpatient molecular heterogeneities of tumors and repertoires of drugs or their molecular targets currently in use in clinical oncology. We estimated molecular heterogeneity using genomic (whole exome sequencing) and transcriptomic (RNA sequencing) data for 4890 tumors taken from The Cancer Genome Atlas database. For thirteen major cancer types, we compared heterogeneities at the levels of mutations and gene expression with the repertoires of targeted therapeutics and their molecular targets accepted by the current guidelines in oncology. Totally, 85 drugs were investigated, collectively covering 82 individual molecular targets. For the first time, we showed that the repertoires of molecular targets of accepted drugs did not correlate with molecular heterogeneities of different cancer types. On the other hand, we found that the clinical recommendations for the available cancer drugs were strongly congruent with the gene expression but not gene mutation patterns. We detected the best match among the drugs usage recommendations and molecular patterns for the kidney, stomach, bladder, ovarian and endometrial cancers. In contrast, brain tumors, prostate and colorectal cancers showed the lowest match. These findings provide a theoretical basis for reconsidering usage of targeted therapeutics and intensifying drug repurposing efforts.

## 1. Introduction

Cancers have high levels of molecular heterogeneity [1]. It is manifested at least at the two levels: *intertumoral*, between different patients with the same type of tumor and *intratumoral*, between different parts of tumor and/or metastases [2]. While intratumoral heterogeneity is thought to be the main cause of drug resistance and relapse of individual cancers, the inter-patient level of heterogeneity hinders the development of universally active anticancer drugs [3,4]. Tumor localization, histologic and molecular differences dictate the need for the development of multiple drugs with different specificities. From the molecular perspective, even tumors of the same localization and histological type are considered a heterogeneous set [5]. The inter-patient heterogeneity of tumors is an important problem in clinical oncology that requires personalization of most of the treatment options [6].

Modern methods in molecular diagnostics enable classification of tumors not only by their histologic type but also by specific genetic features [7]. The information on molecular genetic patterns that determine the course of carcinogenesis, tumor progression and response to treatments is being constantly accumulating [8].

The evolving molecular features of tumors can be evaluated using high-performance genetic, transcriptomic, epigenetic and proteomic assays [9]. The databases of genomic, transcriptomic and epigenetic profiles of both normal and tumor samples have been created; for example, The Cancer Cenome Atlas (TCGA), Oncobox Atlas of Normal Tissue Expression (ANTE), International Cancer Genome Consortium (ICGC), Genotype-Tissue Expression (GTEX) project [10,11,12,13]. 

In a number of large-scale experimental and meta-analysis investigations, multiple cancer molecular biomarkers and critical pathways of carcinogenesis were identified. Understanding the molecular biology of tumor heterogeneity stimulates updating of therapeutic instruments including targeted anticancer drugs. These drugs have a known molecular target, which increases their selectivity and reduces side effects compared to conventional chemotherapy [14,15,16]. For several cancers, target drugs demonstrated dramatic increase of survival [14]. There are currently around 200 anticancer target drugs available in the domain of clinical oncology worldwide [17]. Outstanding improvements were achieved for hematological and lymphoid malignancies, non-small cell lung cancer (NSCLC), colorectal cancer, gastro-intestinal stromal tumors, breast cancer, kidney cancer, melanoma and others [18,19,20,21,22]. Nevertheless, results of clinical trials show that generally targeted therapeutics are strongly effective only for minor cohorts of patients, whereas the average efficacy for all patients of a given cancer type remains relatively low [14,18]. Furthermore, the mechanism of action of targeted drugs dictates restrictions of their use only for the patients with specifically expressed/manifested respective molecular targets or other relevant biomarkers [23]. For example, therapeutic monoclonal antibodies against EGFR, such as Cetuximab and Panitumumab, are poorly effective in *KRAS/NRAS*-mutated colorectal cancers, that is, for ~40% of the patients [24]. Trastuzumab, a therapeutic antibody targeting the HER2 receptor, is effective only for the ~20%–30% cohort of patients overexpressing the *ERBB2* gene [18]. 

The effectiveness of Epidermal Growth Factor Receptor (EGFR)-specific tyrosine kinase inhibitors in NSCLC is associated with mutations in *EGFR* gene, that is, deletions of the 19–21^st^ exons and amplifications of *EGFR* gene positively correlate with the clinical benefit of treatment [20]. In melanoma or NSCLC, specific inhibitors of BRAF kinase Vemurafenib and Dabrafenib and MEK inhibitors Binimetinib and Trametinib are used only in *BRAF*-mutated tumors. Tyrosine kinase inhibitor Larotrectinib is recommended for solid tumors with fusions of *NTRK* genes. In turn, inhibitors of isocitrate dehydrogenase-1 (IDH1) protein are used for the treatment of patients with relapsed or refractory acute myeloid leukemia with a diagnostic mutation in *IDH1* gene. To the date, there are similar molecularly-guided restrictions for more than 50 targeted cancer drugs [25]. 

Moreover, the US Food and Drug Administration (FDA) now recommends developing companion diagnostic tests for all new cancer drugs entering the pharmaceutical market [26]. Several marketed target drugs already have such companion molecular tests [25]. Alternatively, clinicians can use transcriptomics-based high throughput data-driven second opinion systems of targeted therapeutics selection [27,28,29,30,31]. 

Historically, the treatment standards have been formulated for most types of cancer [32,33,34,35,36,37,38,39,40,41,42,43,44]. However, the underlying treatment schemes are focused primarily on localization or histological characteristics of a tumor but do not consider most projections of its molecular phenotype. Moreover, the currently accepted treatment regimens do not reflect the degree of intertumoral heterogeneity within a particular cancer type [1]. Exceptions are made only for a narrow spectrum of specific genetic damages, such as diagnostic mutations discussed above or epigenetic changes like methylation of *MGMT* gene promoter in brain tumors [18,19,24,45]. 

Thus, it can be assumed theoretically that cancer types with higher degree of intertumoral molecular heterogeneity have a smaller proportion of responses to a particular targeted therapy and more different drugs should be accepted for clinical use in these instances. In this study we investigate whether this concept is in line with the currently accepted standards of care in oncology. 

We estimated the degree of intertumoral heterogeneity in different primary cancer localizations by analyzing the whole exome and gene expression data of 4890 tumors taken from TCGA project database. The extent of heterogeneity was assessed by measurements of clustering quality. For all major cancer types, we compared heterogeneities at the levels of mutations and gene expression with the repertoires of targeted therapeutics and their molecular targets accepted by the National Comprehensive Cancer Network (NCCN) guidelines. In total, 85 drugs were investigated that included the classes of targeted monoclonal antibodies; immunotherapeutics; tyrosine kinase, cyclin, histone deacetylase, poly-ADP ribose polymerase and proteasomal inhibitors; rapalogues; antiangiogenic and microtubule agents and the others. Collectively, they covered 82 individual molecular targets. For the first time, we showed that the repertoires of molecular targets of accepted drugs did not correlate with molecular heterogeneities of different cancer types. On the other hand, we found that the current clinical recommendations for the available cancer drugs were strongly congruent with the gene expression but not gene mutation patterns. We detected the best match among the drugs usage recommendations and molecular patterns for the kidney, stomach, bladder, ovarian and endometrial cancers. In contrast, brain tumors, prostate and colorectal cancers showed the lowest match. These findings provide a theoretical basis for reconsidering clinical guidelines and intensifying drug repurposing efforts. 

## 2. Results

### 2.1. Biosample Sets

Intertumoral variation was measured here using gene expression data and mutation frequencies in genes using molecular profiles for 4890 patient biosamples of thirteen cancer types. The following cancer types were investigated (according to TCGA classification): (*i*) Bladder Urothelial Carcinoma, (*ii*) Glioblastoma Multiforme and Lower Grade Glioma, (*iii*) Breast Invasive Carcinoma, (*iv*) Cervical Squamous Cell Carcinoma and Endocervical Adenocarcinoma, (*v*) Colon Adenocarcinoma and Rectum Adenocarcinoma, (*vi*) Kidney Renal Clear Cell Carcinoma and Kidney Renal Papillary Cell Carcinoma, (*vii*) Liver Hepatocellular carcinoma, (*viii*) Lung Adenocarcinoma and Lung Squamous Cell Carcinoma, (*ix*) Ovarian Serous Cystadenocarcinoma, (*x*) Prostate Adenocarcinoma, (*xi*) Stomach Adenocarcinoma, (*xii*) Thyroid Carcinoma, (*xiii*) Uterine Corpus Endometrial Carcinoma. 

For those types of cancer, major variation parameters were compared. However, the variation may depend on the sample size. Ideally, data sets of the same size should be used for reasons of statistical equivalence in comparative analyses. However, in the reality this is difficult to obtain groups of biosamples of the same size for different cancer types. Artificially reducing sizes of most cancer type datasets down to the size of the minimal set is not desirable because it can bias their distribution patterns. Another possibility is taking datasets of not identical but comparable sizes. To this end, we selected from TCGA project database thirteen cancer type datasets each having 136–797 samples, where average number of samples per dataset was 377. For every sample, whole exome and RNA sequencing data were available.

**Figure 1:**
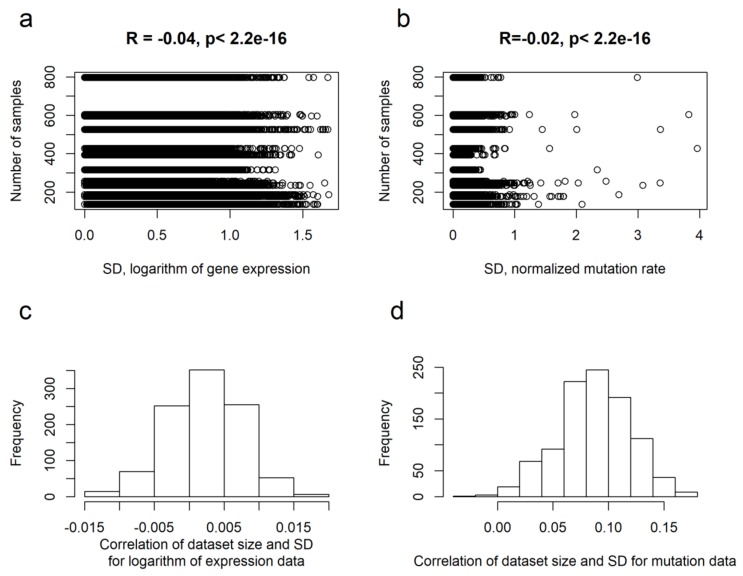
Correlation between molecular variability and sampling size for cancer groups under investigation. Standard deviation (SD) was used as a measure of variation, R is Pearson correlation coefficient. (**a**) Correlation of dataset size and SD for gene expression (logarithmic on base 10). Each dot represents one gene, totally 36304 genes investigated for 13 cancer types. (**b**) Correlation of cancer group size and SD for normalized gene mutation rate. Each dot represents one gene, totally 19618 genes investigated for 13 cancer types. (**c**) Distribution of correlation coefficients between SD and number of samples in a group for 1000 randomly formed datasets of gene expression data (logarithmic on base 10). (**d**) Distribution of correlation coefficients between SD and number of samples in a group for 1000 randomly formed datasets of normalized gene mutation rate.

We then checked whether there is a relationship between the variation of gene expression and the number of biosamples per cancer type. For the expression levels of every gene, we calculated a standard deviation (SD) in every cancer type. Pearson correlation was then calculated for the obtained SDs and the respective numbers of biosamples per cancer type (Figure 1a). Similarly, mutation data were investigated using normalized gene mutation rates and their SDs (Figure 1b) [46]. In both cases, we found no significant correlation between SDs and sample sizes for the thirteen tumor types investigated. Furthermore, we performed randomization computational experiment to investigate the effect of sampling size on our expression and mutation dataset. The groups of biosamples that previously corresponded to cancer types were now selected randomly for 1 000 times. Total numbers of samples per randomized groups were equal to the numbers of samples per group in the initial TCGA cancer datasets (Table 1). 

We then calculated SDs for logarithms of gene expression and for normalized mutation rates and correlated them with the numbers of samples per group (Figure 1c,d). We observed lack of significant correlations between SD and number of samples per group for both mutation and gene expression data. We, therefore, concluded that the cancer group sizes investigated here are acceptable for further functional comparisons of inter-patient heterogeneities. 

### 2.2. Intertumoral Heterogeneities

The inter-patient (intertumoral) heterogeneities were measured for every cancer type in two ways: by similarity with the other cancer types and by similarity between different samples in the same cancer type. 

**Figure 2:**
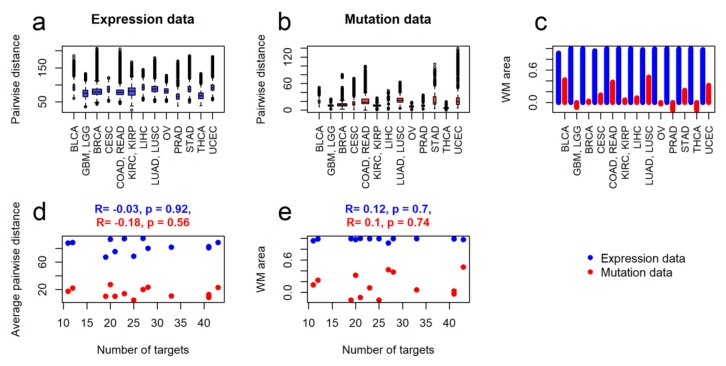
Intragroup tumor heterogeneities of molecular profiles in comparison with number of targets for National Comprehensive Cancer Network (NCCN)-recommended drugs. *Expression data* means logarithmic Deseq2-normalized expression counts, *mutation data* means normalized mutation rate. Measures of heterogeneity: (**a**) pairwise intragroup distances for expression data, (**b**) pairwise intragroup distances for mutation data, (**c**) WM area as indicator of clustering quality for the expression (blue bins) and mutation (red bins) data. (**d**) Correlation of average pairwise distance per group with numbers of molecular targets for the respective NCCN-recommended drugs, for expression and mutation data. (**e**) Correlation of clustering quality (WM area) with number of molecular targets for the respective NCCN-recommended drugs, for expression and mutation data. Cancer type abbreviations: BLCA -Bladder Urothelial Carcinoma, GBM—Glioblastoma multiforme, LGG—Lower Grade Glioma, BRCA—Breast invasive carcinoma, CESC—Cervical squamous cell carcinoma and endocervical adenocarcinoma, COAD—Colon adenocarcinoma, READ—Rectum adenocarcinoma, KIRC—Kidney renal clear cell carcinoma, KIRP—Kidney renal papillary cell carcinoma, LIHC—Liver Hepatocellular carcinoma, LUAD—Lung adenocarcinoma, LUSC—Lung squamous cell carcinoma, OV—Ovarian serous cystadenocarcinoma, PRAD—Prostate adenocarcinoma, STAD—Stomach adenocarcinoma, THCA—Thyroid carcinoma, UCEC—Uterine Corpus Endometrial Carcinoma.

The similarities in the same cancer type were measured for all genes as pairwise distances between either normalized mutation rates or logarithms of expression, Figure 2a,b. We then compared intragroup heterogeneities of different cancer types with numbers of molecular targets for the respective National Comprehensive Cancer Network (NCCN)-recommended targeted therapeutics. We also did cancer stage-specific comparisons for stages I-IV, where only samples and corresponding molecular targets of drugs were considered for each stage (Supplementary File 1). 

To perform cancer stage-specific investigations, we selected matched transcriptomic and mutational profiles for 3211 previously untreated cases with known cancer stage. We investigated only cancer types with a certain stage having 40 and more sample to increase statistical significance of heterogeneity analysis.

For both expression and mutation data, we found no significant correlations between the intragroup pairwise distances and numbers of the respective molecular targets of drugs, both for the whole set of samples (Figure 2d) and for the cancer stage-specific subsets (Supplementary File 1).

The similarities of different cancer types were measured by their abilities to specifically cluster in a general clustering dendrogram of 4890 tumor samples. Samples of more heterogenous cancer types did not form separate clusters and showed mixed patterns with samples from the other cancer types and vice versa. For heterogeneity assessments, cancer types were taken one by one and compared versus mixed samples of all the remaining cancer types. Two sorts of labels were used for the samples, that is, whether they belong to a cancer type under investigation or not. On the dendrogram of 4890 tumor samples (for both expression and mutation data) clustering quality reflects the degree of separation of the cancer type under study from the others, and, consequently, the degree of its intragroup similarity. The quality of clustering was assessed using the Watermelon Multisection (WM) method (Supplementary File 2, [10,13,47,48,49,50,51,52,53,54,55,56]),where a bigger value of a specific metric termed WM area corresponds to a higher intragroup similarity (Figure 2c).It is worth noting that measurements of the WM area suggested an almost perfect separation of every type of cancer from the others using gene expression data (Figure 2c). However, the mutation data generally showed a very mixed pattern between the different cancer types. Nevertheless, the WM area for mutation data was relatively high for three types of cancer—colon/rectum adenocarcinoma, lung adenocarcinoma/squamous cell carcinoma and bladder urothelial carcinoma (Figure 2c). Intriguingly, two of these cancer types (colorectal, lung cancer) are outstanding for their known specific mutation patterns strongly associated with the outcomes of targeted therapies that work for these cancers but not the others [20,24,57]. Finally, from the perspective of quality of clustering, we found lack of correlation between the intragroup similarities of cancer groups and numbers of molecular targets for the respective NCCN-recommended drugs (Figure 2e), for both expression and mutation data. The same phenomenon was observed also for the cancer stage-specific subsets of gene expression and mutation data (Supplementary File 1)

We then repeated these analyses considering only molecular data for the 82 genes that encode molecular targets for the NCCN-recommended drugs in the thirteen cancer types under study. For the same 4890 tumor samples, we established 82-gene expression profiles. For the mutation data, we analyzed only 2696 tumor samples, because the rest had no mutations in the drug target genes and screened only for 78-gene profiles as no mutation data were obtained for all the samples. 

**Figure 3:**
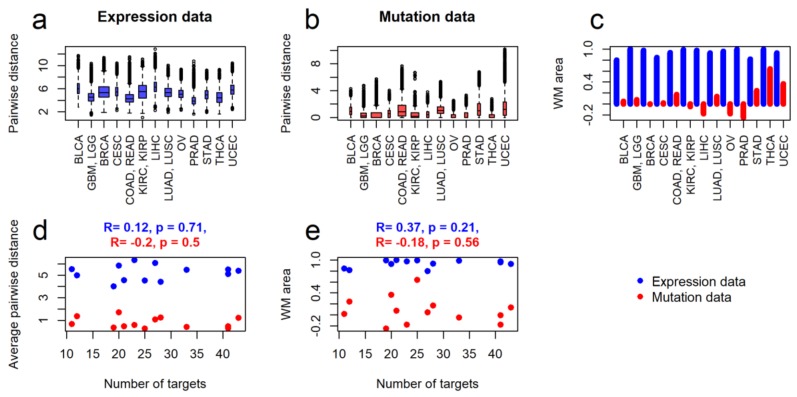
Intragroup tumor heterogeneities of molecular profiles for drug target genes in comparison with number of targets for NCCN-recommended drugs. *Expression data* means logarithmic Deseq2-normalized expression counts, *mutation data* means normalized mutation rate. Measures of heterogeneity: (**a**) pairwise intragroup distances for expression data, (**b**) pairwise intragroup distances for mutation data, (**c**) WM area as indicator of clustering quality for the expression (blue bins) and mutation (red bins) data. (**d**) Correlation of average pairwise distance per group with numbers of molecular targets for the respective NCCN-recommended drugs, for expression and mutation data. (**e**) Correlation of clustering quality (WM area) with number of molecular targets for the respective NCCN-recommended drugs, for expression and mutation data. Cancer type abbreviations: BLCA -Bladder Urothelial Carcinoma, GBM—Glioblastoma multiforme, LGG—Lower Grade Glioma, BRCA—Breast invasive carcinoma, CESC—Cervical squamous cell carcinoma and endocervical adenocarcinoma, COAD—Colon adenocarcinoma, READ—Rectum adenocarcinoma, KIRC—Kidney renal clear cell carcinoma, KIRP—Kidney renal papillary cell carcinoma, LIHC—Liver Hepatocellular carcinoma, LUAD—Lung adenocarcinoma, LUSC—Lung squamous cell carcinoma, OV—Ovarian serous cystadenocarcinoma, PRAD—Prostate adenocarcinoma, STAD—Stomach adenocarcinoma, THCA—Thyroid carcinoma, UCEC—Uterine Corpus Endometrial Carcinoma.

Overall, the latter type of analyses fully confirmed the trends established initially using the whole-exome and transcriptome input data (Figure 3), also for the cancer stage-specific subsets (Supplementary File 3). The major difference was that for the mutation data in target genes, the thyroid cancer showed the highest WM area, thus reflecting the most peculiar target gene mutation profiles for the samples of this group (Figure 3c). This fact can be at least partly explained by outstandingly high frequency of *BRAF V600E* mutation in the most frequent papillary subtype of thyroid cancer. In contrast, bladder cancer samples now showed average value of WM area which may reflect apparently poor response of this cancer to the targeted therapies [57]. However, the lung cancer and colorectal cancer groups of samples as before showed relatively high values of WM area which is in line with the previous considerations. Taken together, our results clearly suggest that for the groups investigated there was no significant correlation for the different cancer types between the intertumoral heterogeneity and number of molecular targets for the cancer therapeutics (Figure 3d,e).

**Figure 4:**
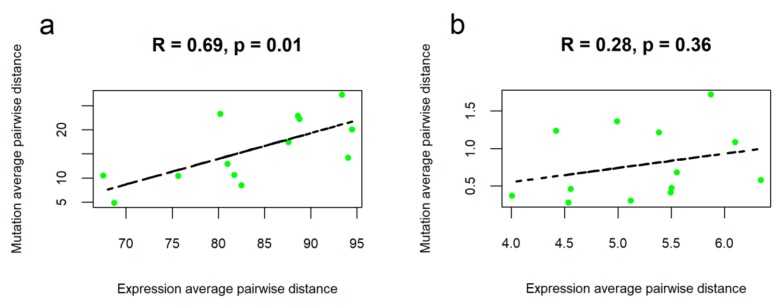
Correlation between cancer type-specific average pairwise distances for gene expression and mutation data. Each dot corresponds to one cancer type, R is Pearson correlation coefficient. Correlations are shown for molecular profiles of all genes (**a**) and for drug target genes (**b**).

Interestingly, we observed statistically significant correlation between cancer type-specific average pairwise distances for the mutation and expression profiles of all genes (Figure 4a). The same trend was seen also for the stage-specific subsets of cancer samples (Supplementary File 4). This finding most probably reflects direct relationship of overall genetic changes and altered gene expression regulation. Bigger number of mutated genes here means overall stronger alteration of the expression profiles. However, our data also suggest that this pattern may have a complex nature and is evident only for the high-scale genetic profiles because there was no significant correlations found for the fraction of 78 drug target genes (Figure 4b, Supplementary File 4).

However, for the individual tumor samples from the same datasets we previously showed no correlation between normalized tumor-specific gene expression changes and normalized mutation rates [58].

### 2.3. Clustering of Cancer Types in Relation with Recommended Targeted Therapeutics and Molecular Data

**Figure 5:**
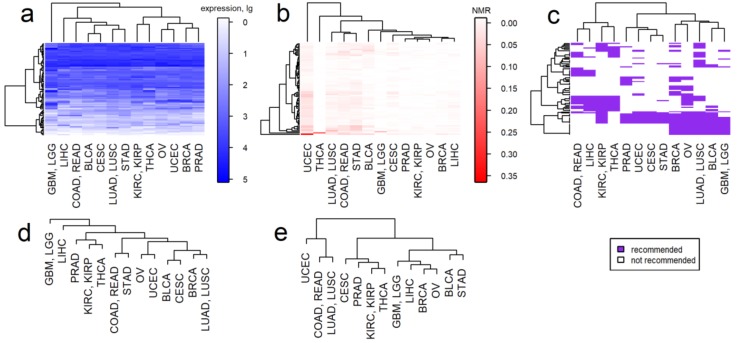
Clustering of thirteen cancer types by molecular profiles and targets of NCCN-recommended drugs. Clustering by (**a**) averaged expression profiles (logarithm of Deseq2 normalized expression counts) for NCCN-recommended drug target genes, (**b**) averaged mutation profiles (NMR) for NCCN-recommended drug target genes, (**c**) molecular targets of NCCN-recommended drugs, (**d**) expression profiles (logarithm of Deseq2 normalized expression counts) for all genes, (**e**) mutation profiles (NMR) for all genes. Cancer type abbreviations: BLCA—Bladder Urothelial Carcinoma, GBM—Glioblastoma multiforme, LGG—Lower Grade Glioma, BRCA—Breast invasive carcinoma, CESC—Cervical squamous cell carcinoma and endocervical adenocarcinoma, COAD—Colon adenocarcinoma, READ—Rectum adenocarcinoma, KIRC—Kidney renal clear cell carcinoma, KIRP—Kidney renal papillary cell carcinoma, LIHC—Liver Hepatocellular carcinoma, LUAD—Lung adenocarcinoma, LUSC—Lung squamous cell carcinoma, OV—Ovarian serous cystadenocarcinoma, PRAD—Prostate adenocarcinoma, STAD—Stomach adenocarcinoma, THCA—Thyroid carcinoma, UCEC—Uterine Corpus Endometrial Carcinoma.

We then clustered molecular profiles averaged for every cancer type. The averaged profiles were calculated separately for every cancer type for both gene expression and mutation data. As before, the clustering was performed in two modes: for all genes and for a reduced set of target genes of NCCN-recommended drugs (Figure 5), including cancer stage-specific clustering, (Supplementary File 5).

Interestingly, clustering of the cancer types by expression data reflected the anatomical proximity of tissues or organs of primary tumor localizations. This trend was more pronounced for the clustering of full expression profiles (Figure 5a,d). In turn, for the mutation-based clustering by both *all* and *drug target* genes, we observed a tendency for colorectal cancer, lung cancer, uterine corpus endometrial carcinoma, bladder urothelial carcinoma and stomach adenocarcinoma to cluster together. These cancers are known for their high frequency of mutations [59]. 

It is worth noting that the kidney, prostate and thyroid cancers strongly tended to cluster together in both mutation and expression-based dendrograms (Figure 5a,b,d,e), including cancer stage-specific subsets (Supplementary File 5). 

The molecular-based clustering was then compared with the clustering by the molecular targets of NCCN-recommended drugs for the particular cancer types (Figure 5c). We assessed whether the cancer types that had similar repertoires of recommended drugs were also clustering together according to molecular profiles and vice versa. We screened clustering features for a mix of cancer stages (Figure 5c) and also separately for all cancer stages I-IV (Supplementary File 5). 

For example, for a mix of cancer stages we observed three major clusters of cancer types according to the target drugs used (Figure 5c). The rightmost cluster differed from the rest by frequent therapeutic targeting of tubulins, PARP, hormone receptors, cyclin dependent kinases and CD molecules. The leftmost cluster had targets such as receptors for vascular, placental, platelet derived and endothelial growth factors, NTRK, DDR2, MAPK11, other tyrosine kinases: ABL1, EPHA2, TEK and FRK. Finally, for the middle cluster including prostate, stomach, endometrial and cervical cancers we observed the minimal number of drug targets (Figure 5b,e). 

This type of analysis was done for every specific cancer stage (I-IV, Supplementary File 5) and for all investigations we assessed whether drug usage clustering was reflected by the clustering according to DNA mutation or gene expression data (Table 2). For some cancer types like kidney, thyroid and colorectal cancers, enough information was available for targeted drugs recommendations in all stages, whereas for the prostate, ovarian, endometrial, cervical and brain tumors there was no enough information of stage-specific targeted drugs usage to enable clustering (Table 2). For both stage-specific and unspecific settings, we quantified coincidences of molecular- and drug usage-based clustering for all cancer types (Table 2). 

We found that clustering by gene expression profiles was significantly more congruent with the drugs recommendation-based clustering rather than clustering by mutation data (82% coincidence for *expression* vs. only 49% for *mutation* data, Table 3). Drug usage in some cancer types was perfectly matching the molecular profiles (e.g., kidney, bladder, stomach, endometrial, cervical and ovarian cancers for *expression* data; kidney, prostate, endometrial, ovarian and brain tumors for *mutation* data). In turn, the matching outsiders were the brain, prostate and colorectal cancers for *expression* data and the lung, colorectal, endometrial and stomach cancers for *mutation* data (Table 3). The kidney, liver, endometrial and ovarian cancers were highly matching with drugs usage profiles for both *expression* and *mutation* data and the colorectal cancer was rather poorly matching for both types of molecular data (Table 3).

## 3. Discussion

Molecular classification of tumors is a promising field in cancer research that can bring new diagnostic, prognostic and therapeutic options along with improved treatment outcomes [60]. High throughput sequencing provides an instrument for modern classification of tumors based on whole exome mutation profiles and/or gene expression data. The classes may be the groups of tumors showing specific molecular features, like mutation and gene expression patterns. The heterogeneity of cancer types depends on the number of molecular classes/subclasses. However, the criteria for cluster allocation are most frequently subjectively determined by the researcher. Heterogeneity of a given type of cancer can be measured by characterizing mixing of the respective samples with the other types on clustering dendrograms. In this case, homogeneous types of cancer will form separate clusters, whereas in heterogenous types samples can be mixed with the other types. However, quantitative assessment of the quality of clustering, especially with high number of samples (e.g., thousands) is a complex non-intuitive task.

For this purpose, a method termed Watermelon Multisection (WM) was developed that enables relatively quick and objective assessment of clustering quality and hence the intertumoral heterogeneity. The method is based on algorithmic assessment of entropy on every cut of clustering. We speculate that WM may be useful also for the analysis of other types of Big Data in biomedicine. In this study, we applied WM to assess heterogeneities of thirteen cancer types presented by 4890 tumor samples with genomic and transcriptomic data. To date, these samples were already described as a heterogeneous group [61,62], but, to our knowledge, their inter-tumor heterogeneity was never assessed numerically. Here we quantitatively characterized intertumoral heterogeneities between and inside different cancer types.

The analysis has been performed independently for the gene expression and DNA mutation data, both at the levels of full set of human genes and for the fraction of drug target genes. For the first time we performed a qualitative assessment of the relationship between intertumoral heterogeneity and repertoires of clinically approved targeted therapeutics. We investigated here the molecular profiles for 4890 patients representing thirteen cancer types and assessed molecular targets of 85 targeted therapeutics. We found that there is no correlation between the intertumoral heterogeneity and the number of recommended therapeutics/drugs molecular targets. Our data demonstrate that currently the repertoire of approved/recommended targeted drugs does not reflect the spectrum of molecular genetic variants of cancers and probably could be expanded in the case of most highly heterogenous cancers. 

On the other hand, clustering of cancers by gene expression data reflects their anatomical and histological proximities, whereas clustering by mutations shows little dependence on these factors (Figure 5). Overall, the most frequently mutated drug target genes were *BRAF* for thyroid cancer, *FGFR2* for endometrial cancer, *EGFR* for brain tumors, *MGMT* for colorectal cancer, *FGFR2* and *FGFR3* for bladder cancer, *NTRK3* for non-small cell lung cancer and *NTRK2*, *FGFR2*, *EGFR* for stomach cancer. These mutations were mentioned in the previous literature but additional studies are necessary to investigate whether they can be considered clinically actionable. Thus, thyroid carcinomas bearing *BRAF* mutations are less sensitive to BRAF inhibitors than melanomas and develop primary or acquired resistance due to additional mutations and activation of alternative signaling pathways that reinforce ERK signaling [63]. Recently NRG Oncology/Gynecologic Oncology Group study showed association of *FGFR2* mutations with poor outcomes in endometrial cancer [64]. Targeting the EGFR signal transduction pathway in brain tumors faces the issue of rapid adaptation through activation of alternative signaling pathways. The role of temozolomide in colorectal cancer still remains controversial and further research is warranted. Temozolomide showed a modest activity in colorectal cancers with *MGMT* promoter methylation and the corresponding clinical trial did not meet its primary end point [65]. Tracing *FGFR3* mutation is currently used for following bladder cancer recurrence but no related therapeutic options became available [66]. Finally, the role of mutated *NTRK3* as target gene for treatment of non-small cell lung cancer is exploited by clinical trials [67], whereas targeting *FGFR2* and *EGFR* in stomach cancer is considered promising for improving current strategies [68].

Our results also add to the discussion of whether gene expression biomarkers have a potential to replace or add value to DNA mutation biomarkers [69]. When comparing the molecular clustering for the drugs recommendation patterns and for the molecular profiles, we found that the gene *expression* data reflected drugs usage practice much better that the gene *mutation* profiles (Table 3). This finding may be in line with the results of a previous clinical investigation WINTHER, where personalized gene expression-guided experimental drug prescriptions to advanced cancer patients resulted in better clinical outcomes than in the case of mutation-based prescriptions [70]. 

NCCN treatment guidelines for drugs usage represent consensus critical assessment of multiple clinical trials results. We showed that they are in line with the transcriptomic features of different cancer types, as reflected by coincidence of clustering by drugs usage patterns and by gene expression in 82% of the cases tested (Table 3). This was not the case for the mutation data, where coincidence was detected in only 49% of the cases, thus strongly arguing that theoretically gene expression data could be more adequate source of information for the cancer treatment selection.

From this perspective, considering the existing repertoire of targeted therapeutics, kidney, stomach, bladder, ovarian and endometrial cancer patients receive the best molecular-matched therapy. In contrast, brain tumor, prostate and colorectal cancer patients receive the least molecular-matched targeted therapy (Table 3). We hypothesize that rethinking drugs usage practice towards more molecularly-matched therapeutics has a potential to improve treatment outcomes in the latter group of cancers.

The best validation for our results could be similar analyses of individual cancer molecular profiles associated with known responses on treatment with targeted therapeutics. Unfortunately, only 130 out of 4890 tumor profiles investigated here have been annotated with known responses to targeted drugs. Such a small number of samples does not make it possible to analyze statistically significant groups of drug responders and non-responders. 

However, further accumulation of clinically annotated high throughput molecular profiles, for example, References [71,72,73] can dramatically improve this situation in the future.

To conclude, we showed here for the first time that the repertoires of current targeted therapeutics do not correspond to molecular heterogeneities of different cancer types. On the other hand, the clinical recommendations for the available cancer drugs are mostly congruent with the gene expression but not gene mutation patterns. We detected the best match among the drugs usage and molecular patterns for the kidney, stomach, bladder, ovarian and endometrial cancers; in contrast, brain tumors, prostate and colorectal cancers showed the lowest match. We propose that studies of a similar design could be carried out periodically to control the progress of anticancer drugs development.

## 4. Materials and Methods 

### 4.1. Tumor Samples

We used molecular profiles for individual tumor samples simultaneously investigated by RNA sequencing and whole exome sequencing in The Cancer Genome Atlas (TCGA) project [10]. For higher statistical significance, we considered only primary localizations having at least one hundred samples with RNA sequencing and exome sequencing data. Totally, 4890 tumor samples were selected for the analysis (Table 1). The term “cancer type” is used as the synonym of cancer from a particular primary site according to TCGA classification [74]. TCGA sample barcodes for all molecular profiles investigated are given in Supplementary Table S1.

For stage-specific studies, we selected molecular profiles for previously untreated tumor samples with known stage diagnosed (Supplementary Table S1). For our analyzes, we considered minimal group size of 40 samples per cancer type per stage. For stage I, 1115 tumor samples were analyzed, 997 samples for stage II, 855 samples for stage III and 244 samples for stage IV, totally 3 211 samples. Structure of tumor datasets for different stages is shown on Supplementary Table S2.

### 4.2. Mutation Data

Gene mutation data were extracted from the COSMIC database v76 containing validated mutations for TCGA tumor samples [75]. We selected non-synonymous somatic mutations of the following mutation types: “substitution-missense”, “deletion-frameshift”, “substitution-nonsense”, “insertion-in frame”, “deletion-in frame”, “insertion-frameshift”, “complex-deletion in frame”, “nonstop extension”, “complex”, “complex-compound substitution”, “complex-frameshift”, “complex-insertion in frame”. Mutation profiles were generated for every tumor sample under investigation using *normalized mutation rates* of 19 618 genes. 

*Normalized mutation rate* (*NMR*) was calculated as follows:

(1) $${\mathrm{NMR}}_{n,g}=\frac{1000*N\;\mathrm{mut}(n,g)}{\mathrm{Length}\;\mathrm{CDS}\;(g)},$$

where *NMR_n,g_* is *NMR* of gene *n* in sample *g*; *N mut(n,g)* is the number of mutations for gene *n* in sample *g*; *Length CDS(n)* is the length of coding DNA sequence (CDS) of gene *n* in nucleotides [27,46]. The gene CDS normalization was done to eliminate a bias caused by different gene lengths [46], calculated NMR values are given in Supplementary Table S3. Averaged NMR profiles for thirteen cancer types under investigation are shown on Supplementary Table S4. 

### 4.3. Gene Expression Data

RNA sequencing data were extracted from the GDC portal in HTSeq counts format [76]. For every gene expression profile, Deseq2 normalization was applied [47]. For further analyses we used logarithms of normalized gene counts. 

### 4.4. Clinical Utility of Drugs and Molecular Targets

We extracted information about targeted therapeutics used in clinical practice for treatment of the cancer types under investigation from the US Food and Drug Administration (FDA) portal [25] and from the National Comprehensive Cancer Network (NCCN) guidelines [32,33,34,35,36,37,38,39,40,41,42,43,44]. The inclusion criterion for a therapeutic was its FDA approval and/or recommendation by the NCCN, category higher than 2B. Only those drugs were included that are accepted for the histotypes presenting in the 4890-tumor sampling for the thirteen cancer types investigated here (Table 1), histotypes of all individual samples are listed in Supplementary Table S5. The matrix drug-disease indicating approval of therapeutics for treatment of the cancer types under analysis is shown on Supplementary Table S6. The information was collected for 164 targeted drugs, of them only 85 are accepted for the thirteen cancer types investigated. The stage-specific usage of drugs for the cancer types under study is shown on Supplementary Table S6. Molecular targets of drugs were identified using public databases DrugBank and Selleckchem [17,77]. In total, we identified 82 unique genes for molecular targets of the drugs selected (Supplementary Table S7).

### 4.5. Clustering Parameters and Quality

The clustering of tumor samples was performed using Ward.D2 method [78] using “euclidian” distance method. The assessment of clustering quality was performed by calculation of Watermelon Multisection (WM) metric. WM is the method characterizing entropy of cluster dendrogram on every cut level. The obtained values are compared with random orders of class labels. The final output value of WM termed *WM Area* reflects the quality of clustering. When the elements of different classes are perfectly separated, the clustering has minimal entropy on every dendrogram cut level and *WM area* is equal to 1. When the elements of different classes are randomly distributed among clusters, *WM area* may vary around zero, also taking negative values. WM scoring demonstrates an advantage over other clustering quality metrics, described in detail in Supplementary File 2. 

### 4.6. Data Presentation

The results were visualized using packages grafics and ggplot2 [79,80].

## Figures and Tables

**Table 1:** Sample structure of molecular dataset used.

Primary Site of Tumor	Number of Samples	TCGA Tumor Abbreviation	Full Name of Tumor, TCGA
Bladder	136	BLCA	Bladder Urothelial Carcinoma
Brain	426	GBM, LGG	Glioblastoma multiforme, Lower Grade Glioma
Breast	797	BRCA	Breast invasive carcinoma
Cervix	177	CESC	Cervical squamous cell carcinoma and endocervical adenocarcinoma
Colorectal	603	COAD, READ	Colon adenocarcinoma, Rectum adenocarcinoma
Kidney	595	KIRC, KIRP	Kidney renal clear cell carcinoma, Kidney renal papillary cell carcinoma
Liver	185	LIHC	Liver Hepatocellular carcinoma
Lung	525	LUAD, LUSC	Lung adenocarcinoma, Lung squamous cell carcinoma
Ovary	315	OV	Ovarian serous cystadenocarcinoma
Prostate	256	PRAD	Prostate adenocarcinoma
Stomach	235	STAD	Stomach adenocarcinoma
Thyroid	393	THCA	Thyroid carcinoma
Uterus	247	UCEC	Uterine Corpus Endometrial Carcinoma

**Table 2:** Similarities of drug usage clustering and molecular clustering for different cancer types.

Cancer Type	All Stages, all Genes		All Stages, Drug Target Genes	
**Cancer type / Drug usage cluster**	**DNA mutation**	**Gene expression**	**DNA mutation**	**Gene expression**
COAD, READ / Cluster 1	−	−	−	+
LIHC / Cluster 1	−	−	+	+
KIRC, KIRP / Cluster 1	+	+	+	+
THCA / Cluster 1	+	+	−	+
PRAD / Cluster 2	+	−	+	+
UCEC / Cluster 2	−	+	−	+
CESC / Cluster 2	+	+	+	+
STAD / Cluster 2	−	+	−	+
BRCA / Cluster 3	+	+	+	+
OV / Cluster 3	+	+	+	+
LUAD, LUSC / Cluster 3	−	+	−	+
BLCA / Cluster 3	+	+	+	+
GBM, LGG / Cluster 3	+	−	+	−
	**Stage I, all genes**		**Stage I, drug target genes**	
LIHC / Cluster 1	+	−	+	+
THCA / Cluster 1	+	+	−	−
COAD, READ / Cluster 1	−	+	−	+
KIRC, KIRP / Cluster 1	+	+	+	+
BRCA / Cluster 2	−	+	−	−
LUAD, LUSC / Cluster 2	−	+	−	−
	**Stage II, all genes**		**Stage II, drug target genes**	
LIHC / Cluster 1	+	+	+	+
THCA / Cluster 1	+	+	−	+
COAD, READ / Cluster 1	−	−	−	−
KIRC, KIRP / Cluster 1	+	+	+	+
BRCA / Cluster 2	−	+	+	+
LUAD, LUSC / Cluster 2	+	+	+	+
STAD / Cluster 2	+	+	−	+
	**Stage III, all genes**		**Stage III, drug target genes**	
LIHC / Cluster 1	+	+	+	+
THCA / Cluster 1	+	+	−	−
COAD, READ / Cluster 1	−	−	+	+
KIRC, KIRP / Cluster 1	+	+	+	+
BRCA / Cluster 2	+	+	+	−
LUAD, LUSC / Cluster 2	−	+	−	+
STAD / Cluster 2	+	+	−	+
BLCA / Cluster 2	−	+	+	+
	**Stage IV, all genes**		**Stage IV, drug target genes**	
THCA / Cluster 1	+	+	−	+
KIRC, KIRP / Cluster 1	+	+	−	+
BLCA / Cluster 2	−	+	+	+
COAD, READ / Cluster 2	−	+	+	+

**Table 3:** Overall match of drug usage clustering and molecular clustering for different cancer types.

Cancer Type	Maximum Possible Score (Max)	Mutation Data, Observed Score (Percentage Share of Max)	Expression Data, Observed Score (Percentage Share of Max)
*COAD, READ*	*10+*	*2+ (20%)*	*6+ (60%)*
*LIHC*	*8+*	*7+ (87.5%)*	*6+ (75%)*
***KIRC, KIRP***	*10+*	*9+ (90%)*	***10+ (100%)***
*THCA*	*10+*	*5+ (50%)*	*8+ (80%)*
***PRAD***	*2+*	***2+ (100%)***	*1+ (50%)*
***UCEC***	*2+*	*0+ (0%)*	***2+ (100%)***
***CESC***	*2+*	***2+ (100%)***	***2+ (100%)***
***STAD***	*6+*	*2+ (33%)*	***6+ (100%)***
*BRCA*	*8+*	*3+ (37.5%)*	*6+ (75%)*
***OV***	*2+*	***2+ (100%)***	***2+ (100%)***
*LUAD, LUSC*	*8+*	*0+ (0%)*	*7% (87.5%)*
***BLCA***	*6+*	*4+ (67%)*	***6+ (100%)***
***GBM, LGG***	*2+*	***2+ (100%)***	*0+ (0%)*
**All cancer types**	**76+**	**37+ (49%)**	**62+ (82%)**

## Supplementary Materials

Supplementary materials can be found at https://www.mdpi.com/1422-0067/21/5/1580/s1. **Supplementary file 1.** Analysis of disparity between inter-patient molecular heterogeneity of all genes and repertoires of target drugs used for different types of cancer, performed separately for cancer stages I-IV. **Supplementary file 2.** Detailed description of Watermelon Multisection method. **Supplementary file 3.** Analysis of disparity between inter-patient molecular heterogeneity of drug target genes and repertoires of target drugs used for different types of cancer, performed separately for cancer stages I-IV. **Supplementary file 4.** Correlation between cancer type-specific average pairwise distances for gene expression and mutation data, calculated separately for cancer stages I-IV. **Supplementary file 5.** Clustering of cancer types under investigation by molecular profiles and targets of NCCN-recommended drugs, performed separately for cancer stages I-IV. **Supplementary Table S1.** TCGA barcodes of the investigated in this study and tumor staging information. **Supplementary Table S2.** Stage-specific structure of tumor sample dataset. **Supplementary Table S3.** Normalized mutation rate profiles for 4890 tumor samples. **Supplementary Table S4.** Averaged NMR profiles for thirteen cancer types under study. **Supplementary Table S5.** Histotypes of TCGA tumor samples, separately for general set and for cancer samples with annotated stages. **Supplementary Table S6.** Drug-disease matrix indicating approval of therapeutics for treatment of the cancer types under analysis, separately for general set and for cancer samples with annotated stages. **Supplementary Table S7.** Molecular targets of drugs investigated in this study.

## Abbreviations

FDA	US Food and Drug Administration
NMRNCCN	Normalized mutation rateNational Comprehensive Cancer Network
TCGA	The Cancer Genome Atlas
WM method	Watermelon Multisection method

## References

[1] Bedard P.L., Hansen A.R., Ratain M.J., Siu L.L. (2013). Tumour heterogeneity in the clinic. Nature.

[2] Jamal-Hanjani M., Quezada S.A., Larkin J., Swanton C. (2015). Translational Implications of Tumor Heterogeneity. Clin. Cancer Res..

[3] Grzywa T.M., Paskal W., Włodarski P.K. (2017). Intratumor and Intertumor Heterogeneity in Melanoma. Transl. Oncol..

[4] Miyauchi T., Yaguchi T., Kawakami Y. (2017). Inter-patient and Intra-tumor Heterogeneity in the Sensitivity to Tumor-targeted Immunity in Colorectal Cancer. Japanese J. Clin. Immunol..

[5] Cusnir M., Cavalcante L. (2012). Inter-tumor heterogeneity. Hum. Vaccin. Immunother..

[6] Turashvili G., Brogi E. (2017). Tumor Heterogeneity in Breast Cancer. Front. Med..

[7] Song Q., Merajver S.D., Li J.Z. (2015). Cancer classification in the genomic era: Five contemporary problems. Hum. Genomics.

[8] Harada S., Arend R., Dai Q., Levesque J.A., Winokur T.S., Guo R., Heslin M.J., Nabell L., Nabors L.B., Limdi N.A. (2017). Implementation and utilization of the molecular tumor board to guide precision medicine. Oncotarget.

[9] Shabani Azim F., Houri H., Ghalavand Z., Nikmanesh B. (2018). Next Generation Sequencing in Clinical Oncology: Applications, Challenges and Promises: A Review Article. Iran. J. Public Health.

[10] Tomczak K., Czerwińska P., Wiznerowicz M. (2015). The Cancer Genome Atlas (TCGA): An immeasurable source of knowledge. Contemp. Oncol. (Poznan, Poland).

[11] International Cancer Genome Consortium T.I.C.G., Hudson T.J., Anderson W., Artez A., Barker A.D., Bell C., Bernabé R.R., Bhan M.K., Calvo F., Eerola I. (2010). International network of cancer genome projects. Nature.

[12] Lonsdale J., Thomas J., Salvatore M., Phillips R., Lo E., Shad S., Hasz R., Walters G., Garcia F., Young N. (2013). The Genotype-Tissue Expression (GTEx) project. Nat. Genet..

[13] Suntsova M., Gaifullin N., Allina D., Reshetun A., Li X., Mendeleeva L., Surin V., Sergeeva A., Spirin P., Prassolov V. (2019). Atlas of RNA sequencing profiles for normal human tissues. Sci. Data.

[14] Padma V.V. (2015). An overview of targeted cancer therapy. BioMedicine.

[15] Yan L., Rosen N., Arteaga C. (2011). Targeted cancer therapies. Chin. J. Cancer.

[16] Joo W.D., Visintin I., Mor G. (2013). Targeted cancer therapy--are the days of systemic chemotherapy numbered?. Maturitas.

[17] Wishart D.S., Feunang Y.D., Guo A.C., Lo E.J., Marcu A., Grant J.R., Sajed T., Johnson D., Li C., Sayeeda Z. (2018). DrugBank 5.0: A major update to the DrugBank database for 2018. Nucleic Acids Res..

[18] Nahta R., Esteva F.J. (2007). Trastuzumab: Triumphs and tribulations. Oncogene.

[19] Chapman P.B., Hauschild A., Robert C., Haanen J.B., Ascierto P., Larkin J., Dummer R., Garbe C., Testori A., Maio M. (2011). Improved Survival with Vemurafenib in Melanoma with BRAF V600E Mutation. N. Engl. J. Med..

[20] Gridelli C., De Marinis F., Di Maio M., Cortinovis D., Cappuzzo F., Mok T. (2011). Gefitinib as first-line treatment for patients with advanced non-small-cell lung cancer with activating epidermal growth factor receptor mutation: Review of the evidence. Lung Cancer.

[21] Druker B.J., Talpaz M., Resta D.J., Peng B., Buchdunger E., Ford J.M., Lydon N.B., Kantarjian H., Capdeville R., Ohno-Jones S. (2001). Efficacy and Safety of a Specific Inhibitor of the BCR-ABL Tyrosine Kinase in Chronic Myeloid Leukemia. N. Engl. J. Med..

[22] Gerber D.E. (2008). Targeted therapies: A new generation of cancer treatments. Am. Fam. Physician.

[23] Sawyers C. (2004). Targeted cancer therapy. Nature.

[24] Tan C., Du X. (2012). KRAS mutation testing in metastatic colorectal cancer. World J. Gastroenterol..

[25] U S Food and Drug Administration Home Page. https://www.fda.gov/.

[26] Principles for Codevelopment of an In Vitro Companion Diagnostic Device with a Therapeutic Product. http://www.fda.gov/Drugs/GuidanceComplianceRegulatoryInformation/Guidances/de.

[27] Zolotovskaia M.A., Sorokin M.I., Emelianova A.A., Borisov N.M., Kuzmin D.V., Borger P., Garazha A.V., Buzdin A.A. (2019). Pathway Based Analysis of Mutation Data Is Efficient for Scoring Target Cancer Drugs. Front. Pharmacol..

[28] Artemov A., Aliper A., Korzinkin M., Lezhnina K., Jellen L., Zhukov N., Roumiantsev S., Gaifullin N., Zhavoronkov A., Borisov N. (2015). A method for predicting target drug efficiency in cancer based on the analysis of signaling pathway activation. Oncotarget.

[29] Buzdin A.A., Zhavoronkov A.A., Korzinkin M.B., Venkova L.S., Zenin A.A., Smirnov P.Y., Borisov N.M. (2014). Oncofinder, a new method for the analysis of intracellular signaling pathway activation using transcriptomic data. Front. Genet..

[30] Buzdin A., Sorokin M., Poddubskaya E., Borisov N. (2019). High-Throughput Mutation Data Now Complement Transcriptomic Profiling: Advances in Molecular Pathway Activation Analysis Approach in Cancer Biology. Cancer Inform..

[31] Poddubskaya E.V., Baranova M.P., Allina D.O., Sekacheva M.I., Makovskaia L.A., Kamashev D.E., Suntsova M.V., Barbara V.S., Kochergina-Nikitskaya I.N., Aleshin A.A. (2019). Personalized prescription of imatinib in recurrent granulosa cell tumor of the ovary: Case report. Mol. Case Stud..

[32] Koh W.-J., Abu-Rustum N.R., Bean S., Bradley K., Campos S.M., Cho K.R., Chon H.S., Chu C., Clark R., Cohn D. (2019). Cervical Cancer, Version 3.2019, NCCN Clinical Practice Guidelines in Oncology. J. Natl. Compr. Cancer Netw..

[33] Mohler J.L., Antonarakis E.S., Armstrong A.J., D’Amico A.V., Davis B.J., Dorff T., Eastham J.A., Enke C.A., Farrington T.A., Higano C.S. (2019). Prostate Cancer, Version 2.2019, NCCN Clinical Practice Guidelines in Oncology. J. Natl. Compr. Cancer Netw..

[34] Benson A.B., Venook A.P., Al-Hawary M.M., Cederquist L., Chen Y.-J., Ciombor K.K., Cohen S., Cooper H.S., Deming D., Engstrom P.F. (2018). NCCN Guidelines Insights: Colon Cancer, Version 2.2018. J. Natl. Compr. Cancer Netw..

[35] Ettinger D.S., Aisner D.L., Wood D.E., Akerley W., Bauman J., Chang J.Y., Chirieac L.R., D’Amico T.A., Dilling T.J., Dobelbower M. (2018). NCCN Guidelines Insights: Non–Small Cell Lung Cancer, Version 5.2018. J. Natl. Compr. Cancer Netw..

[36] Giordano S.H., Elias A.D., Gradishar W.J. (2018). NCCN Guidelines Updates: Breast Cancer. J. Natl. Compr. Cancer Netw..

[37] Nabors L.B., Portnow J., Ammirati M., Baehring J., Brem H., Butowski N., Fenstermaker R.A., Forsyth P., Hattangadi-Gluth J., Holdhoff M. (2017). NCCN Guidelines Insights: Central Nervous System Cancers, Version 1.2017. J. Natl. Compr. Cancer Netw..

[38] Koh W.-J., Abu-Rustum N.R., Bean S., Bradley K., Campos S.M., Cho K.R., Chon H.S., Chu C., Cohn D., Crispens M.A. (2018). Uterine Neoplasms, Version 1.2018, NCCN Clinical Practice Guidelines in Oncology. J. Natl. Compr. Cancer Netw..

[39] Jonasch E. (2019). NCCN Guidelines Updates: Management of Metastatic Kidney Cancer. J. Natl. Compr. Canc. Netw..

[40] Morgan R.J., Armstrong D.K., Alvarez R.D., Bakkum-Gamez J.N., Behbakht K., Chen L.-M., Copeland L., Crispens M.A., DeRosa M., Dorigo O. (2016). Ovarian Cancer, Version 1.2016, NCCN Clinical Practice Guidelines in Oncology. J. Natl. Compr. Canc. Netw..

[41] Ajani J.A., D’Amico T.A., Almhanna K., Bentrem D.J., Chao J., Das P., Denlinger C.S., Fanta P., Farjah F., Fuchs C.S. (2016). Gastric Cancer, Version 3.2016, NCCN Clinical Practice Guidelines in Oncology. J. Natl. Compr. Canc. Netw..

[42] Haddad R.I., Nasr C., Bischoff L., Busaidy N.L., Byrd D., Callender G., Dickson P., Duh Q.-Y., Ehya H., Goldner W. (2018). NCCN Guidelines Insights: Thyroid Carcinoma, Version 2.2018. J. Natl. Compr. Cancer Netw..

[43] Flaig T.W., Spiess P.E., Agarwal N., Bangs R., Boorjian S.A., Buyyounouski M.K., Downs T.M., Efstathiou J.A., Friedlander T., Greenberg R.E. (2018). NCCN Guidelines Insights: Bladder Cancer, Version 5.2018. J. Natl. Compr. Cancer Netw..

[44] Benson A.B., D’Angelica M.I., Abbott D.E., Abrams T.A., Alberts S.R., Saenz D.A., Are C., Brown D.B., Chang D.T., Covey A.M. (2017). NCCN Guidelines Insights: Hepatobiliary Cancers, Version 1.2017. J. Natl. Compr. Canc. Netw..

[45] Nagpal S., Thomas L., Recht S. (2012). Advances in the management of glioblastoma: The role of temozolomide and MGMT testing. Clin. Pharmacol. Adv. Appl..

[46] Zolotovskaia M.A., Sorokin M.I., Roumiantsev S.A., Borisov N.M., Buzdin A.A. (2019). Pathway Instability Is an Effective New Mutation-Based Type of Cancer Biomarkers. Front. Oncol..

[47] Love M.I., Huber W., Anders S. (2014). Moderated estimation of fold change and dispersion for RNA-seq data with DESeq2. Genome Biol..

[48] Forbes S.A., Bhamra G., Bamford S., Dawson E., Kok C., Clements J., Menzies A., Teague J.W., Futreal P.A., Stratton M.R., Haines J.L., Korf B.R., Morton C.C., Seidman C.E., Seidman J.G., Smith D.R. (2008). The Catalogue of Somatic Mutations in Cancer (COSMIC). In Current Protocols in Human Genetics.

[49] Efron B., Tibshirani R. (2007). On testing the significance of sets of genes. Ann. Appl. Stat..

[50] Brock G., Pihur V., Datta S., Datta S. (2008). ClValid: An R package for cluster validation. J. Stat. Softw..

[51] Handl J., Knowles J., Kell D.B. (2005). Computational cluster validation in post-genomic data analysis. Bioinformatics.

[52] Dunn J.C. (1974). Well-Separated Clusters and Optimal Fuzzy Partitions. J. Cybern..

[53] Rousseeuw P.J. (1987). Silhouettes: A graphical aid to the interpretation and validation of cluster analysis. J. Comput. Appl. Math..

[54] Zhu Y., Qiu P., Ji Y. (2014). TCGA-Assembler: Open-source software for retrieving and processing TCGA data. Nat. Methods.

[55] Clifford H., Wessely F., Pendurthi S., Emes R.D. (2011). Comparison of clustering methods for investigation of genome-wide methylation array data. Front. Genet..

[56] Quinlan J.R. (1986). Induction of decision trees. Mach. Learn..

[57] van Kessel K.E.M., Zuiverloon T.C.M., Alberts A.R., Boormans J.L., Zwarthoff E.C. (2015). Targeted therapies in bladder cancer: An overview of in vivo research. Nat. Rev. Urol..

[58] Zolotovskaia M.A., Tkachev V.S., Seryakov A.P., Kuzmin D.V., Kamashev D.E., Sorokin M.I., Roumiantsev S.A., Buzdin A.A. (2020). Mutation enrichment and transcriptomic activation signatures of 419 molecular pathways in cancer. Cancers (Basel)..

[59] Kandoth C., McLellan M.D., Vandin F., Ye K., Niu B., Lu C., Xie M., Zhang Q., McMichael J.F., Wyczalkowski M.A. (2013). Mutational landscape and significance across 12 major cancer types. Nature.

[60] Buzdin A., Sorokin M., Garazha A., Sekacheva M., Kim E., Zhukov N., Wang Y., Li X., Kar S., Hartmann C. (2018). Molecular pathway activation - New type of biomarkers for tumor morphology and personalized selection of target drugs. Semin. Cancer Biol..

[61] Lawrence M.S., Stojanov P., Mermel C.H., Robinson J.T., Garraway L.A., Golub T.R., Meyerson M., Gabriel S.B., Lander E.S., Getz G. (2014). Discovery and saturation analysis of cancer genes across 21 tumour types. Nature.

[62] Lawrence M.S., Stojanov P., Polak P., Kryukov G.V., Cibulskis K., Sivachenko A., Carter S.L., Stewart C., Mermel C.H., Roberts S.A. (2013). Mutational heterogeneity in cancer and the search for new cancer-associated genes. Nature.

[63] Crispo F., Notarangelo T., Pietrafesa M., Lettini G., Storto G., Sgambato A., Maddalena F., Landriscina M. (2019). Braf inhibitors in thyroid cancer: Clinical impact, mechanisms of resistance and future perspectives. Cancers (Basel)..

[64] Jeske Y.W., Ali S., Byron S.A., Gao F., Mannel R.S., Ghebre R.G., DiSilvestro P.A., Lele S.B., Pearl M.L., Schmidt A.P. (2017). FGFR2 mutations are associated with poor outcomes in endometrioid endometrial cancer: An NRG Oncology/Gynecologic Oncology Group study. Gynecol. Oncol..

[65] Westphal M., Maire C.L., Lamszus K. (2017). EGFR as a Target for Glioblastoma Treatment: An Unfulfilled Promise. CNS Drugs.

[66] Couffignal C., Desgrandchamps F., Mongiat-Artus P., Ravery V., Ouzaid I., Roupret M., Phe V., Ciofu C., Tubach F., Mentre F. (2015). The Diagnostic and Prognostic Performance of Urinary FGFR3 Mutation Analysis in Bladder Cancer Surveillance: A Prospective Multicenter Study. Urology.

[67] Rolfo C., Raez L. (2017). New targets bring hope in squamous cell lung cancer: Neurotrophic tyrosine kinase gene fusions. Lab. Investig..

[68] Zhang J., Tang P.M.K., Zhou Y., Cheng A.S.L., Yu J., Kang W., To K.F. (2019). Targeting the Oncogenic FGF-FGFR Axis in Gastric Carcinogenesis. Cells.

[69] Buzdin A., Sorokin M., Garazha A., Glusker A., Aleshin A., Poddubskaya E., Sekacheva M., Kim E., Gaifullin N., Giese A. (2019). RNA sequencing for research and diagnostics in clinical oncology. Semin. Cancer Biol..

[70] Rodon J., Soria J.C., Berger R., Miller W.H., Rubin E., Kugel A., Tsimberidou A., Saintigny P., Ackerstein A., Braña I. (2019). Genomic and transcriptomic profiling expands precision cancer medicine: The WINTHER trial. Nat. Med..

[71] Poddubskaya E.V., Baranova M.P., Allina D.O., Smirnov P.Y., Albert E.A., Kirilchev A.P., Aleshin A.A., Sekacheva M.I., Suntsova M.V. (2018). Personalized prescription of tyrosine kinase inhibitors in unresectable metastatic cholangiocarcinoma. Exp. Hematol. Oncol..

[72] Sorokin M., Poddubskaya E., Baranova M., Glusker A., Kogoniya L., Markarova E., Allina D., Suntsova M., Tkachev V., Garazha A. (2020). RNA sequencing profiles and diagnostic signatures linked with response to ramucirumab in gastric cancer. Cold Spring Harb. Mol. case Stud..

[73] Poddubskaya E., Bondarenko A., Boroda A., Zotova E., Glusker A., Sletina S., Makovskaia L., Kopylov P., Sekacheva M., Moisseev A. (2019). Transcriptomics-Guided Personalized Prescription of Targeted Therapeutics for Metastatic ALK-Positive Lung Cancer Case Following Recurrence on ALK Inhibitors. Front. Oncol..

[74] Wang Z., Jensen M.A., Zenklusen J.C. (2016). A practical guide to The Cancer Genome Atlas (TCGA). Methods in Molecular Biology.

[75] Forbes S.A., Beare D., Boutselakis H., Bamford S., Bindal N., Tate J., Cole C.G., Ward S., Dawson E., Ponting L. (2017). COSMIC: Somatic cancer genetics at high-resolution. Nucleic Acids Res..

[76] Liu J., Lichtenberg T., Hoadley K.A., Poisson L.M., Lazar A.J., Cherniack A.D., Kovatich A.J., Benz C.C., Levine D.A., Lee A.V. (2018). An Integrated TCGA Pan-Cancer Clinical Data Resource to Drive High-Quality Survival Outcome Analytics. Cell.

[77] Selleckem.com. https://www.selleckchem.com.

[78] Murtagh F., Legendre P. (2014). Ward’s Hierarchical Agglomerative Clustering Method: Which Algorithms Implement Ward’s Criterion?. J. Classif..

[79] Wickham H. (2009). Ggplot2 : Elegant graphics for data analysis.

[80] R Core Team (2018) R: A language and environment for statistical computing. https://www.r-project.org/.

