# Supplementary material for: Disparity between Inter-Patient Molecular Heterogeneity and Repertoires of Target Drugs Used for Different Types of Cancer in Clinical Oncology
Source: Int J Mol Sci. 2020 Feb 26;21(5):1580. doi: 10.3390/ijms21051580 (PMC7084891; doi:10.3390/ijms21051580)
Supplement: Supplementary file 1 [file ijms-21-01580-s001.zip › ijms-691043-supplementary/Supplementary File 1AB.docx]

**Supplementary File 1. Analysis of disparity between inter-patient molecular heterogeneity of all genes and repertoires of target drugs used for different types of cancer, performed separately for cancer stages I-IV.**


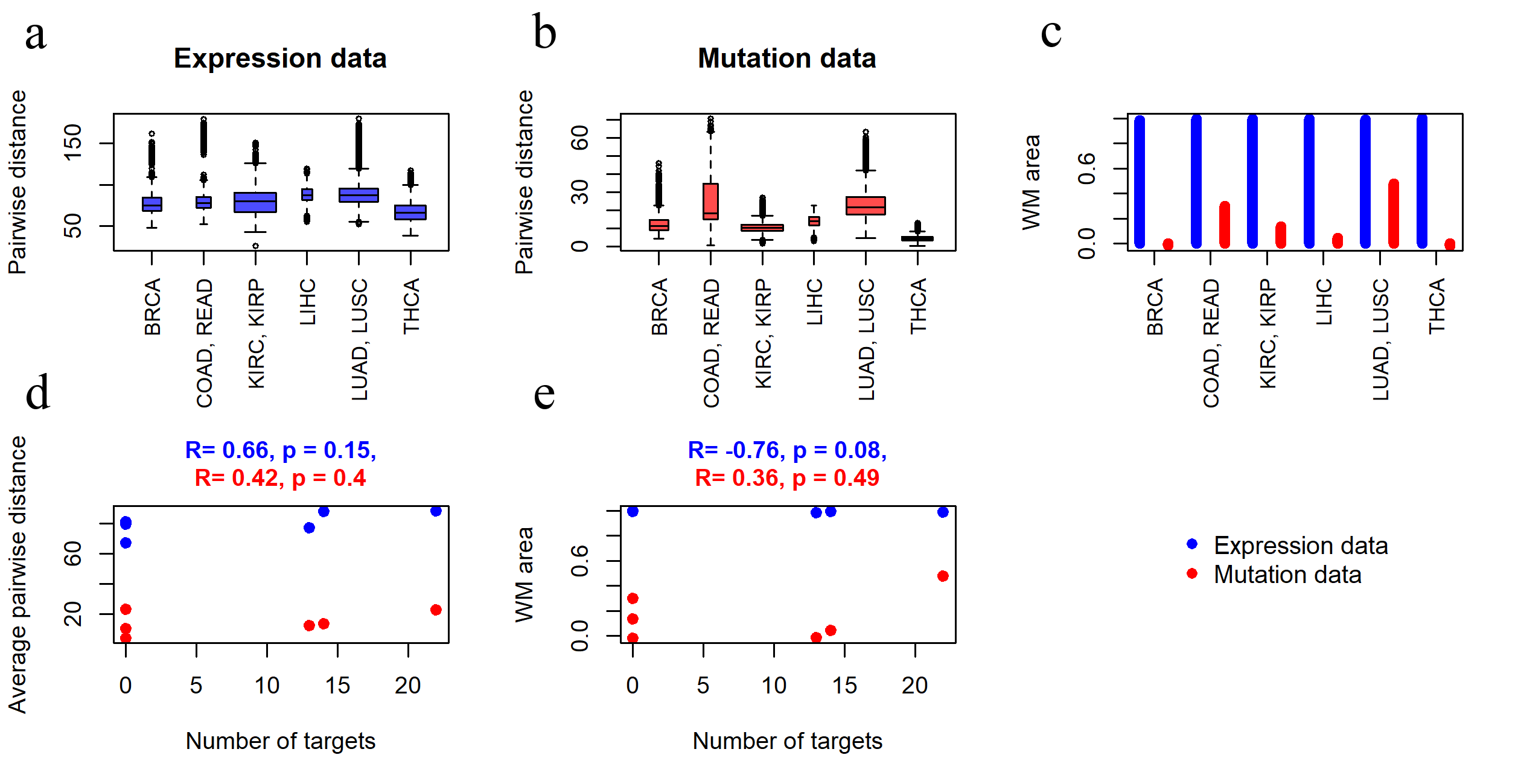


**Figure S1.** Intragroup tumor heterogeneities of molecular profiles in comparison with number of targets for NCCN-recommended drugs in stage I cancers. Expression data means logarithmic Deseq2-normalized expression counts, mutation data means normalized mutation rate. Measures of heterogeneity: (a) pairwise intragroup distances for expression data, (b) pairwise intragroup distances for mutation data, (c) WM area as indicator of clustering quality for the expression (blue bins) and mutation (red bins) data. (d) Correlation of average pairwise distance per group with numbers of molecular targets for the respective NCCN-recommended drugs, for expression and mutation data. (e) Correlation of clustering quality (WM area) with number of molecular targets for the respective NCCN-recommended drugs, for expression and mutation data. Cancer type abbreviations: BRCA - Breast invasive carcinoma, COAD - Colon adenocarcinoma, READ - Rectum adenocarcinoma, KIRC - Kidney renal clear cell carcinoma, KIRP - Kidney renal papillary cell carcinoma, LIHC - Liver Hepatocellular carcinoma, LUAD - Lung adenocarcinoma, LUSC - Lung squamous cell carcinoma, THCA - Thyroid carcinoma.


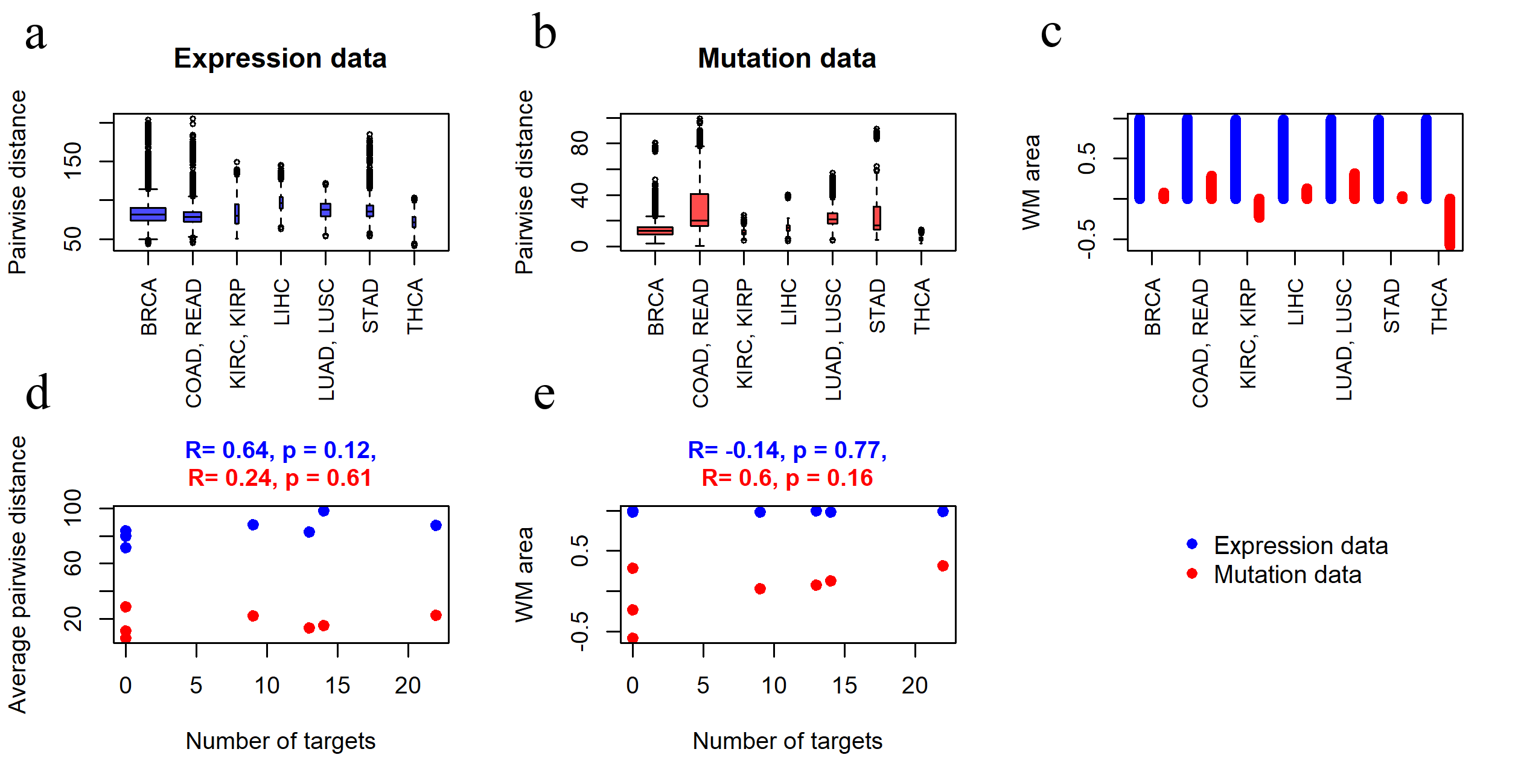


**Figure S2.** Intragroup tumor heterogeneities of molecular profiles in comparison with number of targets for NCCN-recommended drugs in stage II cancers. Expression data means logarithmic Deseq2-normalized expression counts, mutation data means normalized mutation rate. Measures of heterogeneity: (a) pairwise intragroup distances for expression data, (b) pairwise intragroup distances for mutation data, (c) WM area as indicator of clustering quality for the expression (blue bins) and mutation (red bins) data. (d) Correlation of average pairwise distance per group with numbers of molecular targets for the respective NCCN-recommended drugs, for expression and mutation data. (e) Correlation of clustering quality (WM area) with number of molecular targets for the respective NCCN-recommended drugs, for expression and mutation data. Cancer type abbreviations: BRCA - Breast invasive carcinoma, COAD - Colon adenocarcinoma, READ - Rectum adenocarcinoma, KIRC - Kidney renal clear cell carcinoma, KIRP - Kidney renal papillary cell carcinoma, LIHC - Liver Hepatocellular carcinoma, LUAD - Lung adenocarcinoma, LUSC - Lung squamous cell carcinoma, STAD - Stomach adenocarcinoma, THCA - Thyroid carcinoma.


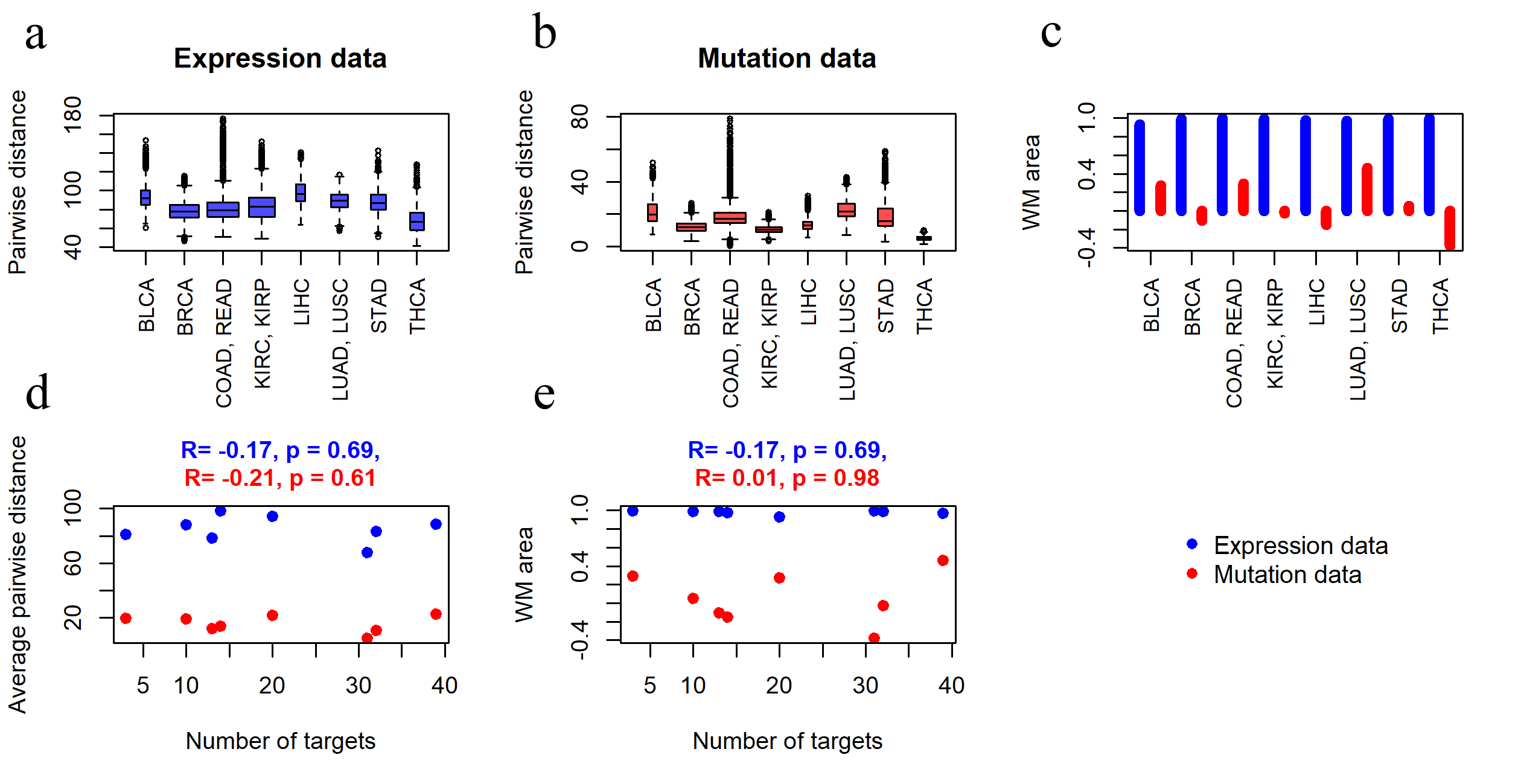


**Figure S3.** Intragroup tumor heterogeneities of molecular profiles in comparison with number of targets for NCCN-recommended drugs in stage III cancers. Expression data means logarithmic Deseq2-normalized expression counts, mutation data means normalized mutation rate. Measures of heterogeneity: (a) pairwise intragroup distances for expression data, (b) pairwise intragroup distances for mutation data, (c) WM area as indicator of clustering quality for the expression (blue bins) and mutation (red bins) data. (d) Correlation of average pairwise distance per group with numbers of molecular targets for the respective NCCN-recommended drugs, for expression and mutation data. (e) Correlation of clustering quality (WM area) with number of molecular targets for the respective NCCN-recommended drugs, for expression and mutation data. Cancer type abbreviations: BLCA -Bladder urothelial carcinoma, BRCA - Breast invasive carcinoma, COAD - Colon adenocarcinoma, READ - Rectum adenocarcinoma, KIRC - Kidney renal clear cell carcinoma, KIRP - Kidney renal papillary cell carcinoma, LIHC - Liver Hepatocellular carcinoma, LUAD - Lung adenocarcinoma, LUSC - Lung squamous cell carcinoma, STAD - Stomach adenocarcinoma, THCA - Thyroid carcinoma.


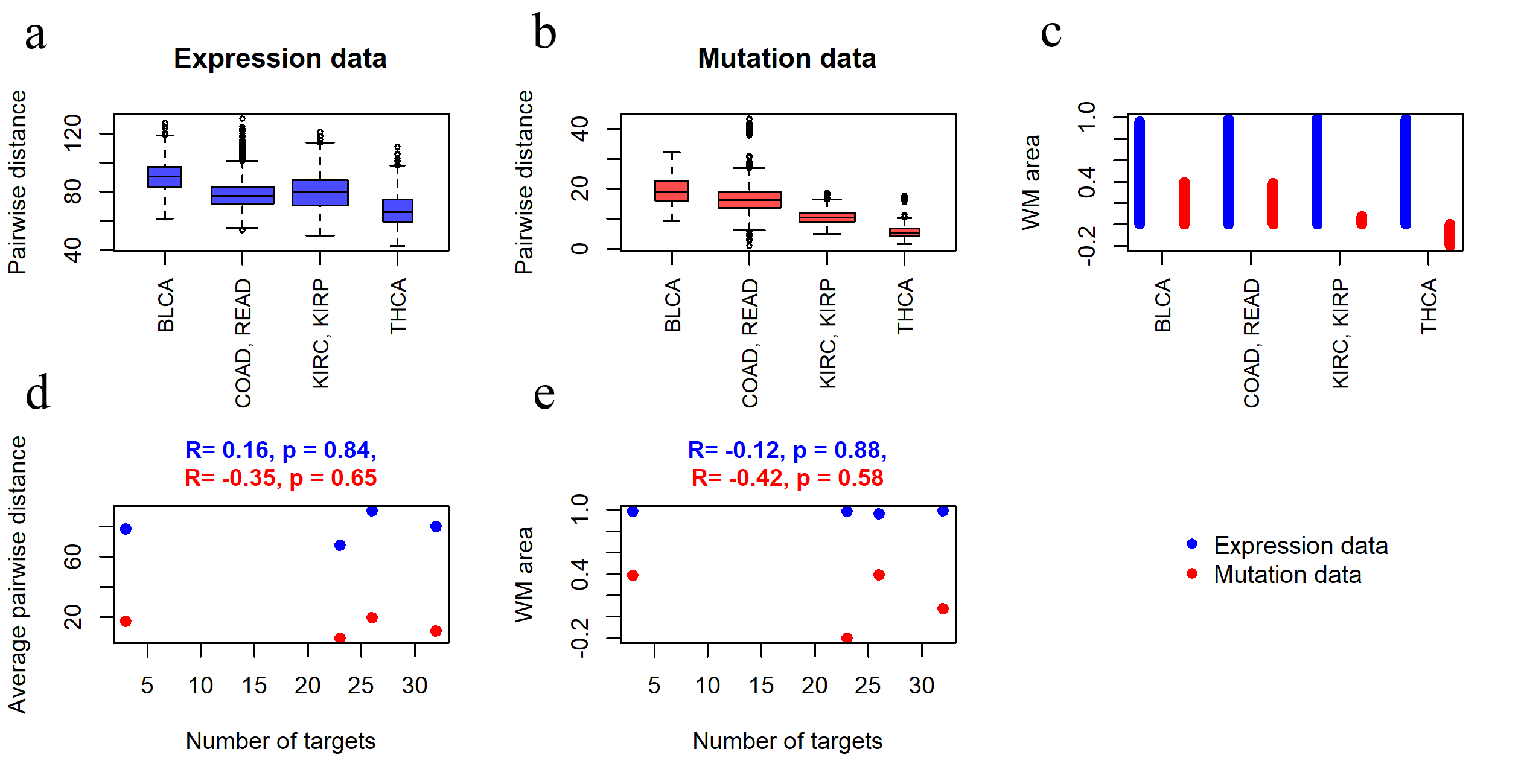


**Figure S4.** Intragroup tumor heterogeneities of molecular profiles in comparison with number of targets for NCCN-recommended drugs in stage IV cancers. Expression data means logarithmic Deseq2-normalized expression counts, mutation data means normalized mutation rate. Measures of heterogeneity: (a) pairwise intragroup distances for expression data, (b) pairwise intragroup distances for mutation data, (c) WM area as indicator of clustering quality for the expression (blue bins) and mutation (red bins) data. (d) Correlation of average pairwise distance per group with numbers of molecular targets for the respective NCCN-recommended drugs, for expression and mutation data. (e) Correlation of clustering quality (WM area) with number of molecular targets for the respective NCCN-recommended drugs, for expression and mutation data. Cancer type abbreviations: BLCA -Bladder urothelial carcinoma, COAD - Colon adenocarcinoma, READ - Rectum adenocarcinoma, KIRC - Kidney renal clear cell carcinoma, KIRP - Kidney renal papillary cell carcinoma, THCA - Thyroid carcinoma.
