# Supplementary material for: Disparity between Inter-Patient Molecular Heterogeneity and Repertoires of Target Drugs Used for Different Types of Cancer in Clinical Oncology
Source: Int J Mol Sci. 2020 Feb 26;21(5):1580. doi: 10.3390/ijms21051580 (PMC7084891; doi:10.3390/ijms21051580)
Supplement: Supplementary file 1 [file ijms-21-01580-s001.zip › ijms-691043-supplementary/Supplementary File 2AB.docx]

**Supplementary File 2. Watermelon multisection: a method for multi-purpose clustering quality assessment**

Due to its intuitive visualization, unsupervised nature and deterministic outcome, hierarchical clustering is often a method of choice for analysis of gene expression/mutation data. However, in its present form this method has an important limitation of manual curation of the output graphical data, thus complicating its use for Big Data analyses. To solve this problem, a method termed Watermelon Multisection (WM) was proposed. WM is the entropy-based method for assessing performance of hierarchical clustering dendrograms relative to a trait of interest, e. g. sample classes. This method evaluates the strength of data association with the trait of interest, describing the entire clustering tree with a metric used to characterize its quality. Using the WM method, numerous dendrograms based on the different feature selections, distance functions or tree building algorithms, can be easily compared disregarding any subjective influence. This is the method for purely computational quality assessment of hierarchical clustering according to matching with pre-defined classes. WM can be of a special usefulness for the analysis of Big Data in life sciences. Its performance in real-world systems biomedicine applications are demonstrated here using RNA-sequencing data from the open-access database The Cancer Genome Atlas (TCGA), and the experimental dataset with multiple normal human tissues.

***The validation datasets.***

***Tumor tissue molecular data***

We extracted gene expression and whole exome DNA mutation profiles from The Cancer Genome Atlas (TCGA) project database ^1^, available at <http://cancergenome.nih.gov/>. For the analysis, we took only the cancer samples having matched both expression and mutation data. We took 50 cancer sample profiles for each of the following 16 tumor types, totally 800 profiles: bladder urothelial carcinoma (BLCA), breast invasive carcinoma (BRCA), cervical squamous cell carcinoma and endocervical adenocarcinoma (CESC), colon adenocarcinoma (COAD), glioblastoma multiforme (GBM), kidney renal clear cell carcinoma (KIRC), kidney renal papillary cell carcinoma (KIRP), brain lower grade glioma (LGG), liver hepatocellular carcinoma (LIHC), lung adenocarcinoma (LUAD), lung squamous cell carcinoma (LUSC), ovarian serous cystadenocarcinoma (OV), prostate adenocarcinoma (PRAD), skin cutaneous melanoma (SKCM), stomach adenocarcinoma (STAD), thyroid carcinoma (THCA).

All the gene expression profiles were obtained using Illumina HiSeq 2000 platform and processed using the RSEMv2 algorithm. The expression data were primarily filtered (genes with total count numbers less than the number of samples were removed from the analysis) and then joined together and normalized using DESeq2 ^2^ software.

The mutation profiles for the same tumor samples were extracted from the COSMIC ^3^ database and then normalized on gene coding sequence lengths to obtains gene mutation enrichment scores.

***Normal tissue data***

159 gene expression profiles for the normal human tissue samples were extracted from the ANTE database (Suntsova et al., 2019a), GSE120795 dataset in Gene Expression Omnibus (GEO) repository. The following expression profiles were taken (tissue type/number of samples): adrenal gland/6; bladder/5; brain/9; colon/12; esophagus/8; kidney/8; liver/8; lung/8; mammary gland/5; normal peripheral blood CD138-positive cells/11; merged group of ovarian, cervical and uterus samples/10; pancreas/8; prostate/6; skeletal muscle/6; skin/6; small intestine/9; stomach/15; thyroid gland/6; tonsil/7, whole blood nuclear cells/6.

**Watermelon Multisection (WM) metric calculation**

Let us consider a set of biological samples with biologically different origin (e. g. derived from different tissue types). Suppose these samples were profiled for high throughput molecular data, e. g. gene expression and mutations data. Here, we refer the different biological origin of samples as *sample class*.

We denote each gene expression/mutation profile *i* = 1, … , *N* with the *F*-dimensional feature vector *X_i_* = (*x*^1^, *x*^2^, … , *x^F^* ), and a corresponding *class label* as *y_i_* ∈ {1, … , *C*}, when *C* is the number of classes. Let *D* = {{*X*_1_, *y*_1_}, {*X*_2_, *y*_2_}, …, {*X_N_*, *y_N_* }} be the whole dataset under investigation.

Any hierarchical clustering method arranges these profiles according to pairwise distances *d*(*X_i_*, *X_j_*), *i* = 1, … , *N*, *j* = 1, …, *i*. Our goal is to evaluate the biological relevance for this arrangement, according to the trait of class labels *Y* = (*y*_1_,…, *y_N_* ). Let us consider the clustering dendrogram for the dataset *D* (see Figure 1[,](#_bookmark0) a). For each fork of the dendrogram, from higher partition levels to lower (i. e. upside down), we analyze the partition set *S_l_* = {$s_{1}^{l}$,… , $s_{K_{l}}^{l}$ } for the dendrogram at the level *l* that corresponds to this fork. Here $s_{k}^{l}$, , *k* = 1, … , *K_l_* is a set of class label values (*y*) for all samples found in the cluster *k* at the fork level *l*.

*k*


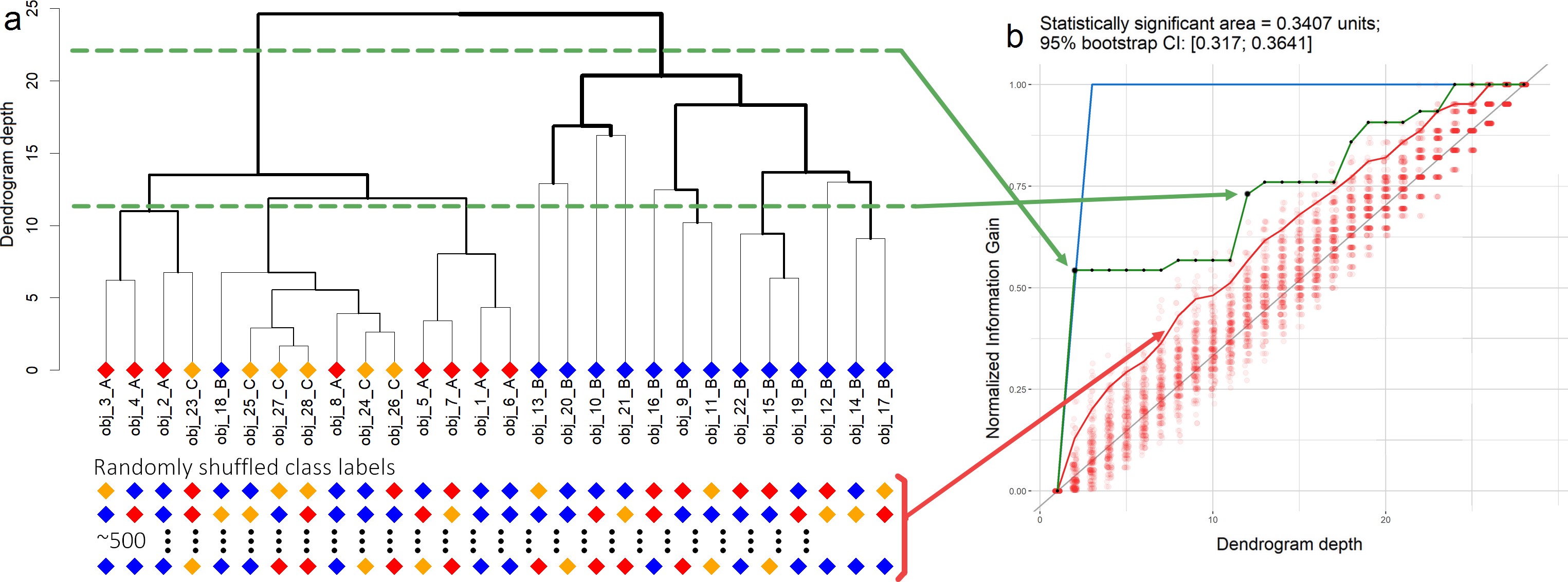


**Figure 1.** Representation of a typical clustering dendrogram (**a**), and corresponding *watermelon plot* (**b**). This figure shows how dendrogram with original labels is transformed into an observed trajectory. Green arrows from panel **a** to panel **b** show the most informative cuts corresponding to information gain surges when the class separation quality improves significantly. Green polygonal chain in the panel **b** follows the information gain trend. Blue polygonal chain in then panel **b** indicates the upper limit for information gain.

Note that at any fork level on the dendrogram, when moving upside down, one of clusters is divided into two lesser clusters (Figure 1). Let us analyze what *information gain (IG)* can be achieved at each fork, from higher levels to lower, compared to the topmost level where all samples belonged to only one cluster.

The *entropy* for any class label set $s_{k}^{l}$ can be found as follows:

$$H\left( Y \right)=-\sum_{i=0}^{C} p_{i}\log p_{i},$$

where *C* is a number of unique classes in class vector *Y* and *p_i_* is a frequency of class *i* in a cluster *k* at the fork level *l*.

The IG provided by this partition is defined as follows ^5^:

$${IG}_{l}\left( S \right)=H\left( Y \right)-\sum_{k=1}^{K_{l}} \frac{n_{k}^{l}}{N}H\left( Y_{k}^{l} \right),$$

where $n_{k}^{l}$ is the number of samples in the cluster *k* for the whole dataset partition at level *l*, and *N* equals to the whole number of samples in the dataset *D*

The example how each fork of a dendrogram provides *information gain* is shown in Figure 2.


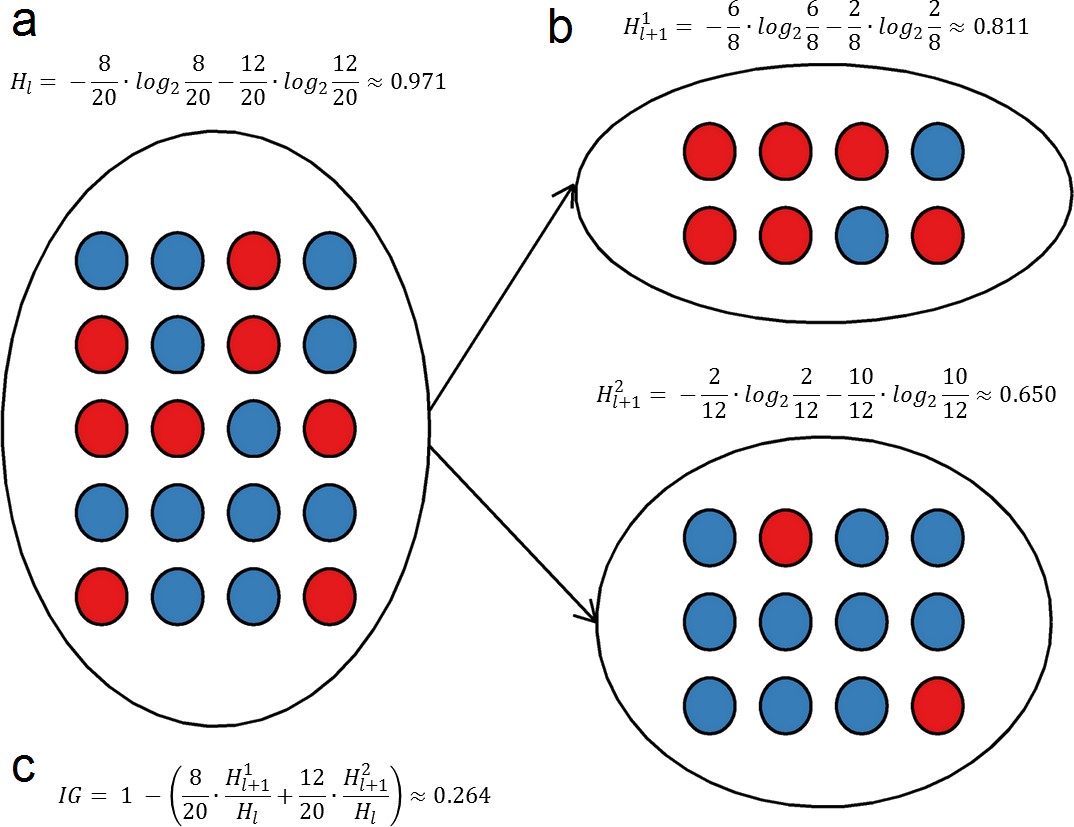


**Figure 2.** Information gain provided by one fork (at the level *l*) of the dendrogram for a dataset with only two classes of samples. **a**: Entropy, *H_l_*, of a cluster before splitting. **b**: A splitting act divides this cluster to two fragments with entropies $H_{l+1}^{1}$ and $H_{l+1}^{2}$. Samples from the datasets belong to either *red* and *blue* classes. **c**: Information gain from the splitting act.

The IG value has upper and lower limits corresponding to the best and the worst scenarios. The *worst case* is when the order of classes among clusters is purely random or there’s an equal number of representatives of each class in every cluster, but that’s very unlikely to occur by chance. The *best case* is when largest classes are initially separated into their own clusters at upper-most cuts.

To obtain a relative value, the IG is normalized by initial entropy:

*IGn_l_* (*S*) = *IG_l_* (*S*)/*H*(*Y*).

The worst *IGn* value is 0, corresponding to equal class percentages in all the clusters, and the best value is 1, which means each cluster contains entities of one class only. Each cluster’s influence is normalized by its size, to prevent bias by unequal size clusters.

Calculating *IGn_l_* (*S_l_*) at every dendrogram fork levels *l* = 1, …, *L* produces an information gain trajectory (Figure 1[,](#_bookmark0) **b**, green curve). It starts at *IGn*_1_ = 0 as non-separated data have full initial entropy by definition, and ends at *IGn_L_* = 1 since at the lowest level each cluster contains only one sample, and the entropy for any set with the only element is zero. This trajectory is steep for well-separated classes as cluster uniformity (by class) increases for good splits, and flatter for a weak or random separation (Figure 1[,](#_bookmark0) **b**, green curve).

In addition, we construct a baseline trajectory $\tilde{IGn}$, which is generated by the null hypothesis *H*_0_: data structure has no statistical association with class labels *y_i_*. This baseline addresses the following two issues:

1. without an adequate comparison (null hypothesis), it’s impossible to estimate quality of observed clustering;
2. entropy-based measures tend to take larger values for factors with multiple levels.

The baseline is obtained by independently shuffling the class labels *Y* for *M* times, in this study — few hundred times, thus generating a set of permuted class labels: *P* = {$\tilde{Y_{1}}$, …, $\tilde{Y_{M}}$}. Then information gain trajectory $\tilde{{IGn}_{m}}$ is calculated for each permuted vector $\tilde{Y_{m}}$, creating a multitude of cases with purely random performance.

Thus, at each dendrogram cut level l we obtain a distribution of empirical random information gain values, coming from the same profiles and distances between them, but with completely shuffled class labels for profiles.

The upper limit for information gain (see the blue polygonal chain in the panel **b** of Figure 1) at level *l* is expressed as difference between the initial entropy and conditional entropy with for partition $S_{l}^{*}$with *l* − 1 biggest classes placed into separate clusters and therefore having a conditional entropy of 0, since information gain explicitly accounts for relative cluster size, thus favoring bigger uniform clusters to emerge first.

To assess significance, at each level *l* we need statistical test for *IGn_l_* to check whether the *q*-value is less than 0.05 relative to a random distribution. Hence, for each *l* we calculate 95-percentile $\tilde{{IGn}_{95}}$ of random information gain values $\tilde{{IGn}_{m}}\left( S_{l} \right)$ *m* = 1, …, *M*. For convenience, we call $\tilde{{IGn}_{95}}$ a *null trajectory* (Figure 1, **b**, red line).

Now we define *watermelon (WM) area*^[[1]](#footnote-1)^ as normalized difference between the observed and null trajectories:

$$WM\left( S,Y \right)=\sum_{l=1}^{L} \frac{IGn\left( S_{l} \right)-\tilde{{IGn}_{95}}\left( S_{l} \right)}{IGn\left( S_{l}^{*} \right)-\tilde{{IGn}_{95}}\left( S_{l}^{*} \right)}.$$

The larger is this difference, the stronger evidence is observed against *H*_0_ (Figure 1[,](#_bookmark0) **b**). It’s worth noting that elements of this difference might be negative, reducing overall performance score when clustering performs worse than best random cases.

***Bootstrap confidence interval***

To assess uncertainty, which arises solely from random shuffles, a bootstrap confidence interval ^6^ is calculated by sampling the random trajectories with replacement and re-calculating the metric. More classes in *Y* require more random trajectories to build a reliable metric, as an absolute area becomes smaller and has higher variation when normalized with respect to lower and upper limits.

Bootstrap is a classical statistical procedure ^6^ used to calculate empirical confidence interval for a parameter if its sampling distribution is unknown, but has a finite variation.

First of all, *M* bootstrap resamples *b_i_* of size *N* are produced by sampling the generated permuted class vectors $\tilde{Y_{m}}$, with replacement which is equal to a set of random trajectories they produce: *B* = {*b*_1_, …, *b_M_*}.

Each random curve is generated separately by shuffling *Y*, so it is correct to assume that they’re independent and identically distributed, *i.e.* satisfy bootstrap requirements. Since these curves have lower and upper limits of 0 and 1, respectively, the watermelon area’s sampling distribution is guaranteed to have finite variance.

This bootstrap curve, *B*, is used to estimate the sampling distribution of watermelon area and calculate confidence interval for this statistic. It is also useful for determining the typical number of random trials required to reach specific certainty threshold to minimize calculation time.

***Visual interpretation of the watermelon plot***

The rationale of the watermelon plot visualization (see Figure 1[,](#_bookmark0)5) is to illustrate how good dendrogram separates classes in contrast to randomly assigned labels. It also shows information gain at every specific level of a dendrogram’s structure. The *x*-axis ranges from 1 to *N* and reflects a depth of the cut; *y*-axis is continuous and shows normalized information gain, ranging from 0 to 1. Sudden increases in information gain uncover most informative splits on dendrogram.

The baseline depends greatly on the number of classes in a dataset. Intuitively, it’s more probable to obtain the equal percentage of two classes in a cluster by chance than for three or more classes, especially if they aren’t equally represented in the data. The information gain is non-decreasing function, and trend shape is more convex when the number of classes grows. Imagine a gradual increase in the number of classes from 2 to *N* — the red curve will gradually shift towards the upper corner.

*Alternative metrics for clustering quality*

The WM metric performance in evaluating clustering quality was compared with three previous methods implemented in R package *clValid* ^7^. Among them, *connectivity* signifies, to what extent the neighbor samples are placed in the same cluster. Define *nn_i_*_(_*_j_*_)_ as the *j-*th nearest neighbor of observation *i*, and let z*nn_i_*_(_*_j_*_)_ be zero if *i* and *nn_i_*_(_*_j_*_)_ are in the same cluster and 1/*j* otherwise. Then, for partition *S* = {*S*_1_,…, *S_C_*} of *N* samples into *C* clusters, the connectivity value ^8^ equals to

$$Conn\left( S \right)=\sum_{i=1}^{N} \sum_{j=1}^{N'} {znn}_{i(j)},$$

where $N^{'}$ is a parameter that determines the number of neighbors that contribute to the connectivity measure. The connectivity can vary between zero and ∞ and is lower for partitions with many small clusters and higher for partitions with large clusters, so that for comparison with WM metric we used the inverse value,

*Inv*_*Conn*(*S*) = 1/*Conn*(*S*).

The *Dunn index* ^9^ is the ratio of the smallest distance between observations not in the same cluster to the largest intra-cluster distance. It is calculated as

$$Dunn\left( S \right)=\frac{\min_{s_{k},s_{l}\in S,s_{k}\neq s_{l}} \left( \min_{i\in s_{k}, j\in s_{l}} d(X_{i},X_{j}) \right)}{\max diam(s_{m})},$$

where *diam*(*s_m_*) is the maximum distance between observations in cluster *s_m_*. The Dunn index can vary between zero and ∞, and it decreases with the number of clusters.

The *Silhouette width* ^10^ is the average of each observation’s silhouette value. The silhouette value measures the degree of confidence in the clustering assignment of a particular observation, with well-clustered observations having values near 1 and poorly clustered observations having values near −1. Silhouette width is expressed by the formula

$$Silh\left( S \right)=\underset{i=1,\ldots,N}{\mathrm{mean}} \left( \frac{b_{i}-a_{i}}{\max\left( b_{i},a_{i} \right)} \right),$$

where *a_i_* is the average distance between the profile *X_i_* and other profiles in the same cluster, ab *b_i_* is the average distance between *X_i_* and profile of the in the *nearest neighboring cluster*.

***Assessment of cancer type similarities using gene expression and DNA mutations data***

The watermelon area (WM) can be used to measure difference between the classes of interest. We used this WM property to build the similarity hierarchy of different cancer types. The WM metric accounts for cluster uniformity in terms of pre-defined element classes. It should be mentioned that many cancer types are intrinsically heterogeneous and some subgroups within one cancer type can be more similar to representatives of another type. It has a consequence that due to the greedy nature of the hierarchical clustering algorithms, these subgroups may be attached to different cancer types. If this is the case, it will be penalized by the information gain score, thus reducing the overall cluster uniformity until tree dissection doesn’t separate this cluster, proportional to the depth on which it is finally joined. Thus, information gain is directly related to separability of heterogenous subtypes, not attributing increased similarity in the same way that class centroids would do, i.e. shift the centroid proportionally to the outlier displacement.

To study cancer type similarities using the WM metric, we performed the analysis of high throughput molecular data obtained in The Cancer Genome Atlas (TCGA) project ^1,11^. Totally, 800 matched RNA-sequencing and whole-exome-mutation profiles were investigated for sixteen cancer types.

Pairwise hierarchical clustering was performed for the log-transformed data subsets obtained after measuring WM areas. Specifically, we took all possible pairs of cancer types, totally 120 possible profile selections for both expression and mutation dataset, each selection having only two types (50 samples of each type).

Watermelon areas were then calculated using 300 random trials for 120 expression and 120 mutation clustering trees. A dissimilarity matrix was then constructed using the pairwise watermelon area. This dissimilarity matrix was used for clustering visualization, thus producing a similarity structure for the different cancer types (Figure 3).


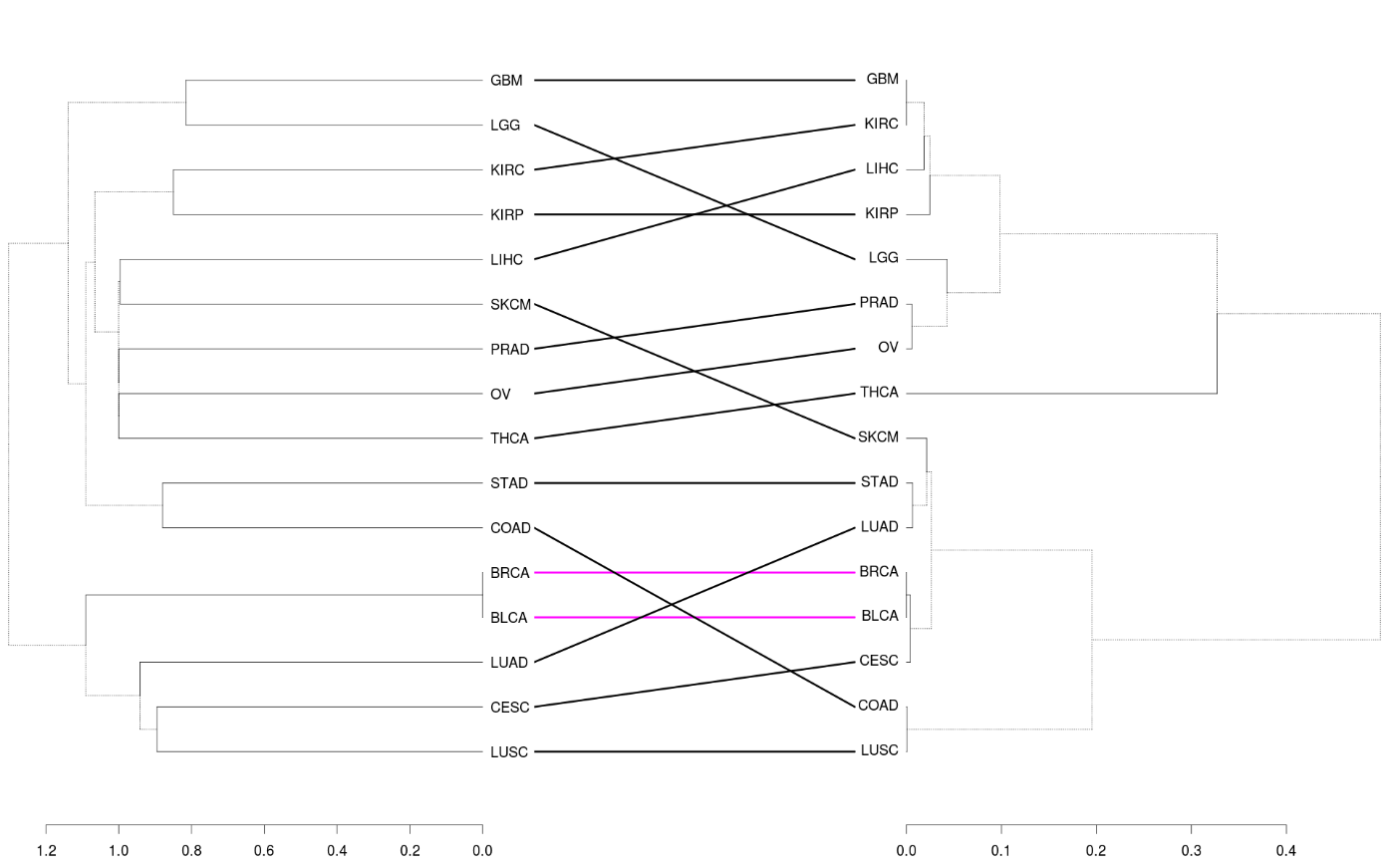
Cancer type similarities according to gene expression and mutation data are shown in Figure 3. Taking apart fundamental features like anatomical proximities of primary tumor localizations and cell type composition similarities, this direct comparison implies that mutation landscape is far less informative in distinguishing the cancer types compared to the gene expression profiles.

**Figure 3.** Comparison of two similarity structures. Left: based on gene expression data, right: based on mutation data. On the *x*-axis, cumulative between – cluster variance is shown. Subtrees contained in both cases are shown in magenta.

Based on mutation hierarchy, the cancer types were more similar than at the level of gene expression. The tree structures for expression and mutation data were remarkably different. For the expression data, the anatomically proximate tumor types clustered together (as for the kidney, breast, lung and brain tumors), whereas this was not the case for the mutation data-driven dendrogram, except for the breast cancer types (Figure 3). An overall compatibility of WM data for mutation and expression profiles was poor (Figure 4).


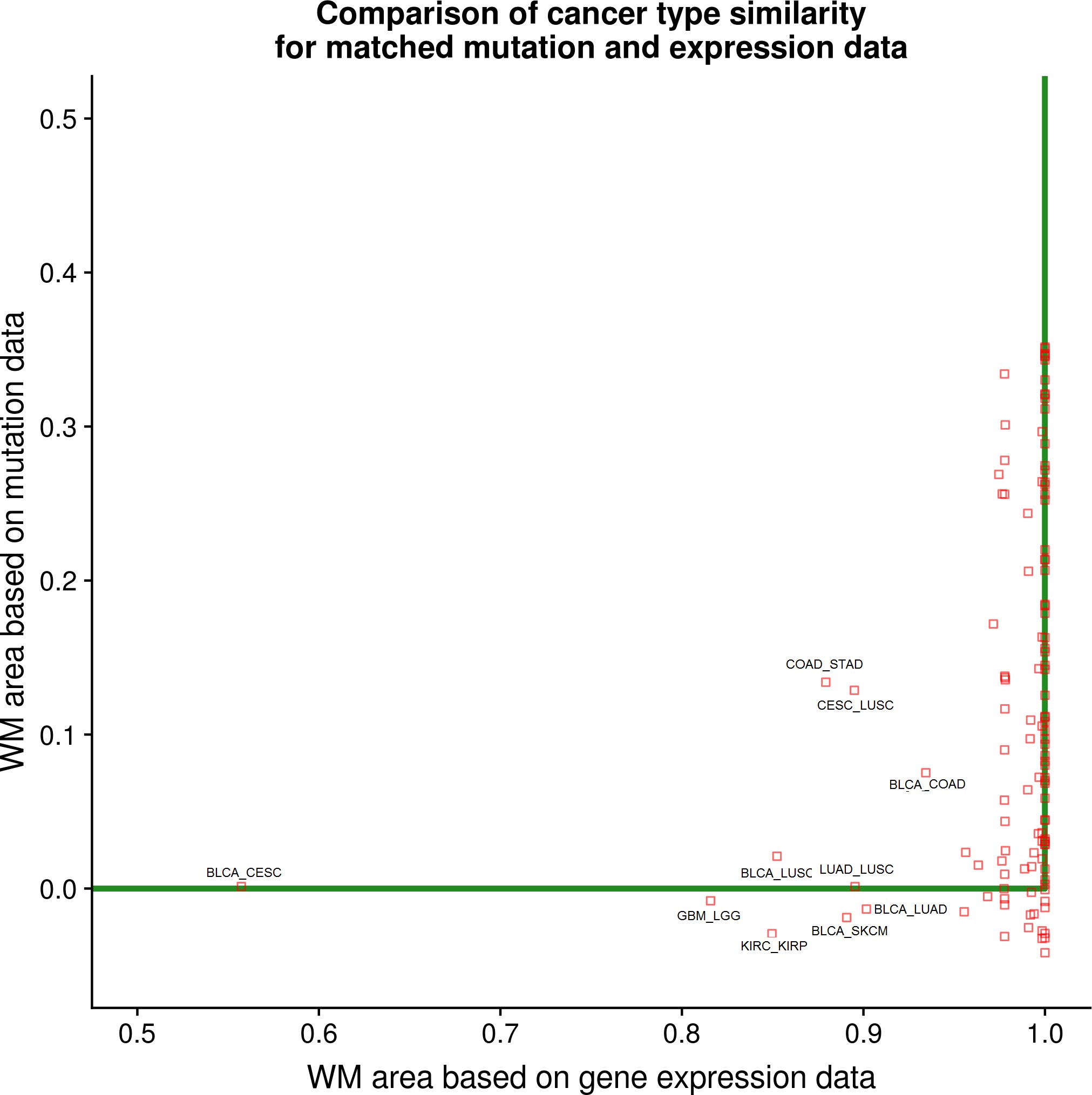


**Figure 4.** Pairwise watermelon areas, expression vs. mutation data. Green borders are sides of a unit square, some cancer types are separated randomly, thus producing negative watermelon area using mutation data. Names of outlier pairs are shown on the graph.

The difference in performance of mutation and expression data-based similarity clustering is exemplified in Figure 5 for LUAD and LUSC tumor types. DNA mutation data showed very poor separation capacity compared to the expression data that had almost perfect trajectory.


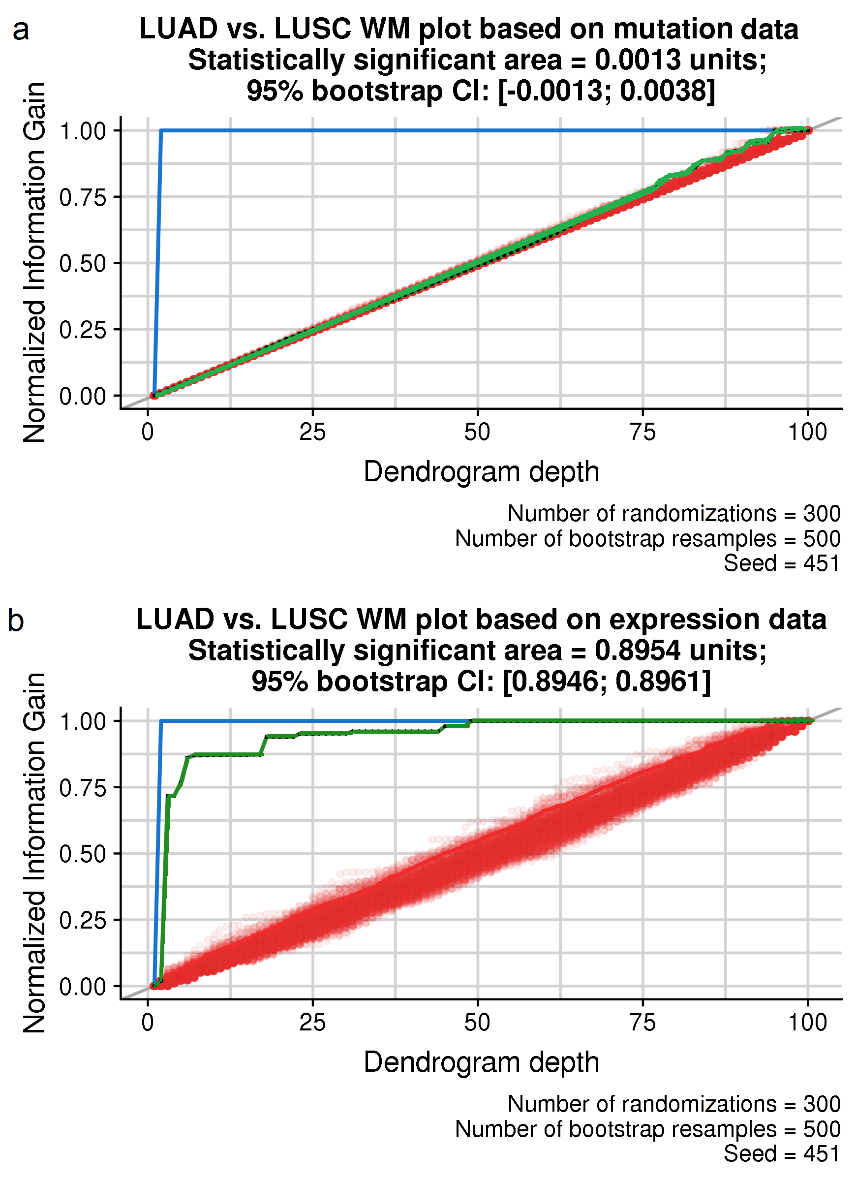


**Figure 5.** WM plots built using mutation (**a**) and expression (**b**) data for discrimination of LUAD and LUSC tumor types.

**Comparison of WM and alternative clustering quality metrics from clValid package**

We compared WM metric with the previous clustering quality metrics, such as inverse connectivity (*Inv*_*Conn*) ^8^, Dunn index (*Dunn*) ^9^, and Silhouette width (*Silh*) ^10^ implemented in the R package *clValid* ^7^. This has been done using 120 expression data dendrograms and 120 mutation data dendrograms from the pairwise TCGA cancer type comparison described above. For each of these dendrograms, which contained only two classes of samples, we calculated the three metrics from *clValid* package and compared them with the performance of WM metric. Figure 6 shows that WM metric doesn’t correlate with any of the above previous metrics, thus confirming its originality.

We then compared performances of the WM and *clValid* metrics (Figure 7). Dendrograms with maximal and minimal values of clustering quality metrics were compared in the following model. A good quality clustering metric should be high for dendrograms with well separated profiles for different class labels and low for dendrograms where profiles for different class labels are shuffled. We observed that only the WM metric performed good (Figure 7) for both gene expression (**a**, **b**) and DNA mutation (**c**, **d**) data. Among other metrics, only the *Silhouette width* satisfied this requirement for the mutation profiles (**o** and **p**), but not for the gene expression data (**m** and **n**).

Similarly, Figure 8 displays the distributions of Euclidean distances between expression and mutation profiles. For a good clustering quality metrics, such distances between the profiles of the same sample type (red graphs) should be essentially lower than between the profiles of different sample types (green graphs), when the metric is high thus suggesting good sample type separation. In contrast, when the metric is low, the differences between the within-class (cyan graphs) and cross-class (violet graphs) distances should be also low. In agreement with previous findings, only the WM metric could satisfy this condition among the four methods tested for both gene expression (**a**) and mutation (**b**) data. This test was failed by the *Inverse connectivity* (**c**, **d**) and *Dunn index* (**e**, **f**) metrics for both data types, and by the *Silhouette width* for gene expression data (**g**).


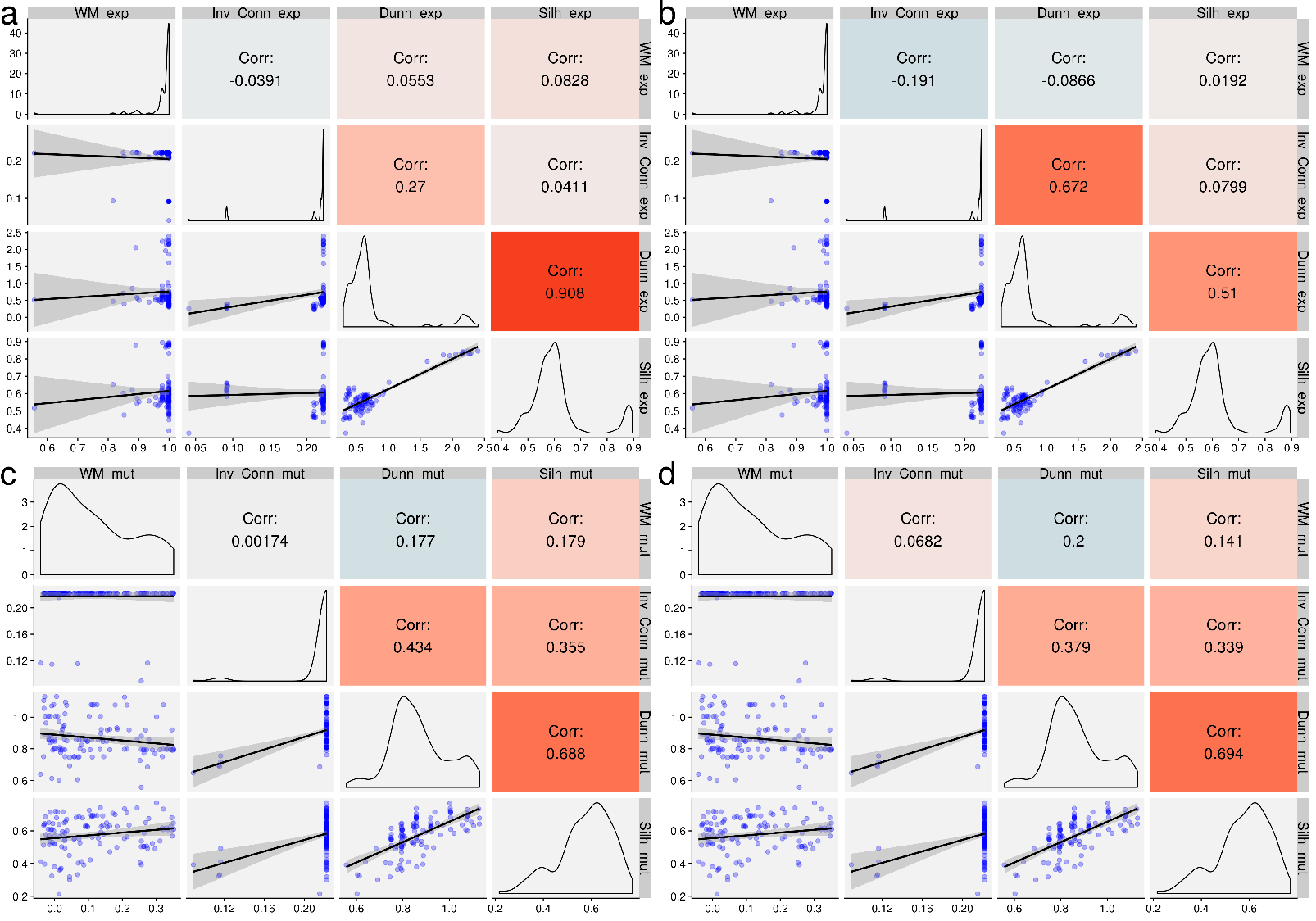


**Figure 6.** Pearson (**a**, **c**) and Spearman (**b**, **d**) pairwise correlations between four clustering quality metrics (*W M*, *Inv*_*Conn*, *Dunn* and *Silh*) for expression (**a**, **b**) and mutation (**c**, **d**) TCGA profiles. Each metric was calculated for each dendrogram that contained expression/mutation profiles for a selected pair of cancer types from the TCGA dataset. In every panel (**a** – **d**) the main diagonal icons show probability densities for metric values. The icons below the main diagonal display scatter plot comparisons of the respective pairs of quality assessment metrics. The icons above the main diagonal show correlations for the respective comparisons.


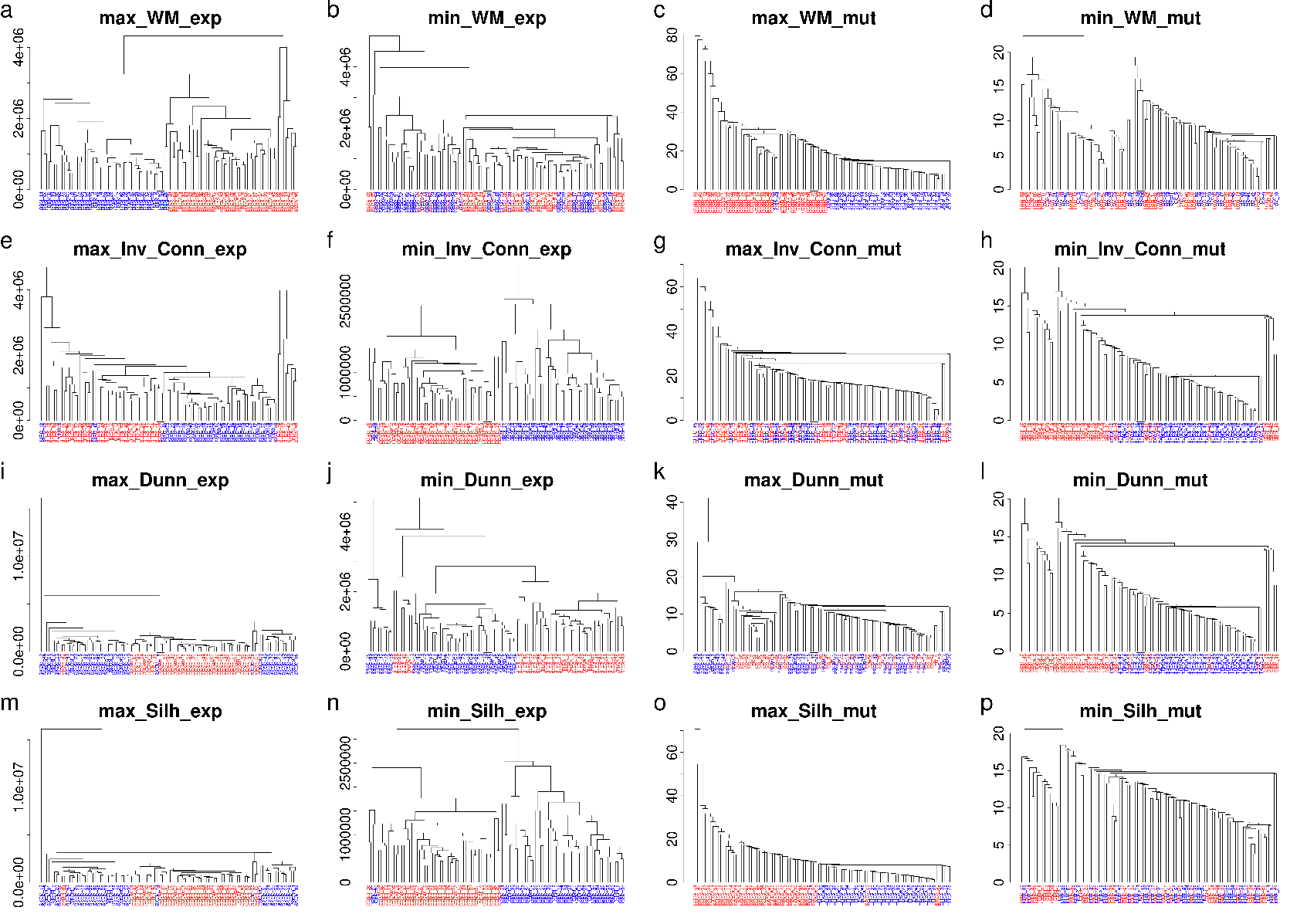


**Figure 7.** Clustering dendrograms for pairwise profile class separation. The pairs of tumor types were selected according to the maximal (**a**, **c**, **e**, **g**, **i**, **k**, **m**, **o**) or minimal (**b**, **d**, **f**, **h**, **j**, **l**, **n**, **p**) values of the respective clustering quality metrics (*W M*: **a**, **b**, **c**, **d**; *Inv*_*Conn*: **e**, **f**, **g**, **h**; *Dunn*: **i**, **j**, **k**, **l**; *Silh*: **m**, **n**, **o**, **p**). Results shown for the gene expression data: panels **a**, **b**, **e**, **f**, **i**, **j**, **m**, **n**. Results shown for the mutation data: panels **a**, **b**, **e**, **f**, **i**, **j**, **m**, **n**.


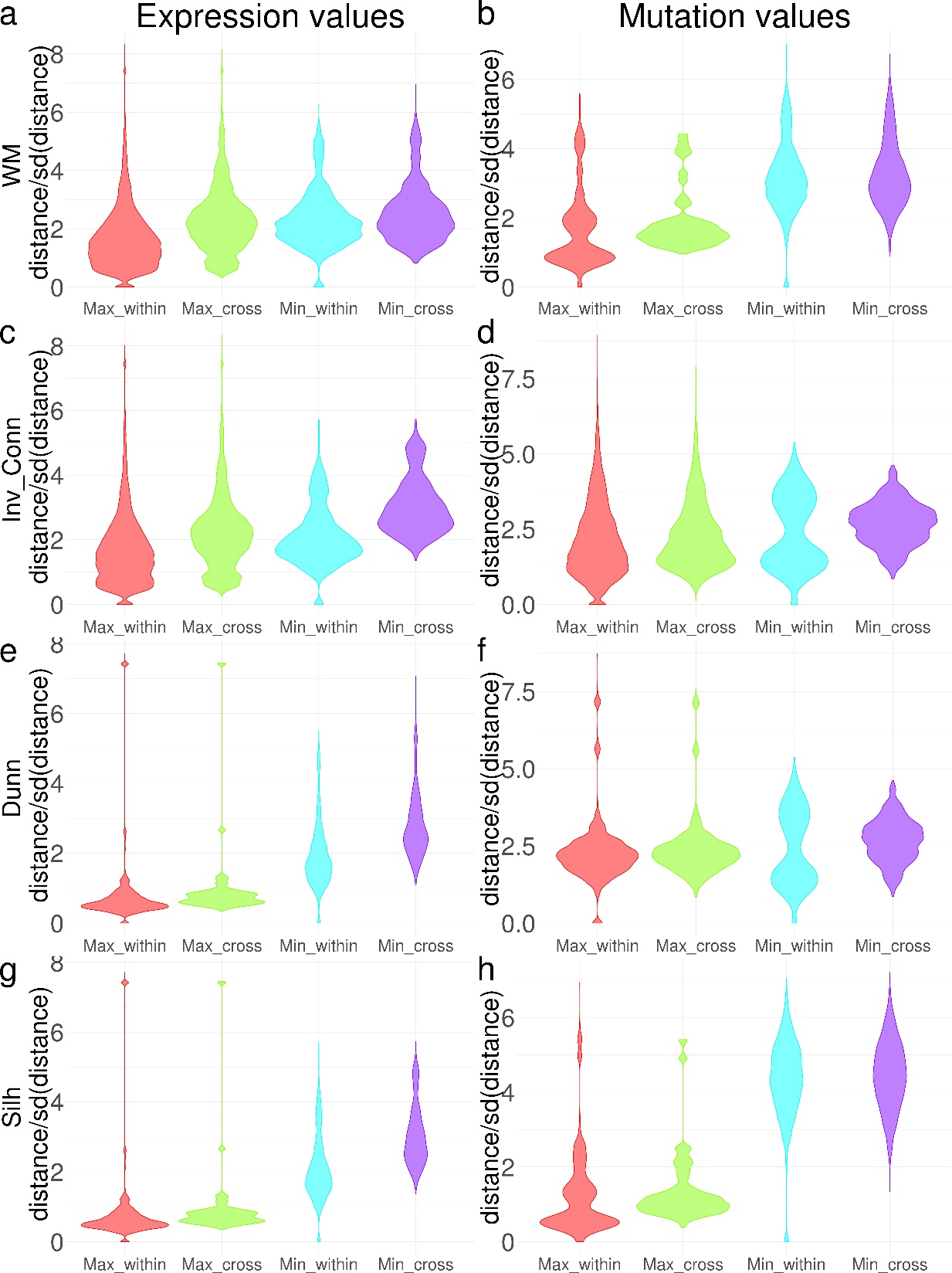


**Figure 8.** Euclidean distances between profile vectors for pairwise class separation tests. Red graphs: dendrograms with maximum metric value, within one cancer type. Green graphs: dendrogram with maximum metric value, between two cancer types. Cyan graphs: dendrograms with minimal metric value, within one cancer type. Violet graphs: dendrograms with minimal metric value, between two cancer types. Gene expression profiles: **a**, **c**, **e**, **g**. Mutation profiles: **b**, **d**, **f**, **h**. *W M* metric: **a**, **b**. *Inv*_*Conn* metric: **c**, **d**. *Dunn* metric: **e**, **f**. *Silh* metric: **g**, **h**.

***WM analysis for 20 types of normal human tissues by expression data***

We then used WM analysis for RNA-sequencing data corresponding to 159 human healthy tissue samples from ANTE database ^4^ representing 20 tissue types. Prior to the analysis, the samples were normalized with respect to size factors ^2^. To prevent data skewness, we used log-transformation of normalized profiles.

We investigated capacities of different distance metrics and clustering algorithms to form groups corresponding to the biological nature of biosamples, i. e. according to the tissue type. Genes with lower numbers of reads are generally considered less reliable and less informative than those having higher number of reads ^2^. We thus formed subsets of all 100% (q100) genes, 80% of genes with higher read numbers (q80), q60, q40, q20 and q0, where q0 represented 0.99% of genes with the highest number of reads.

We tested all agglomerative algorithms with monotonously increasing distance, namely: *average*, *complete*, *mcquitty*, *single*, *ward.D* and *ward.D2* ^12^. For each combination of the above parameters we calculated WM area with 500 random iterations; bootstrap confidence intervals were obtained using 500 resamples. By trying these parameters, we could choose the best performing clustering method according to WM area. For the given model *single* was systematically the worst algorithm, whereas *ward.D* and *ward.D2* methods outperformed all the others for all the feature sets (Figure 9). The examples of best (WM area ≈ 0.73) and worst (WM area ≈ 0.32) dendrograms are shown on Figure 10.


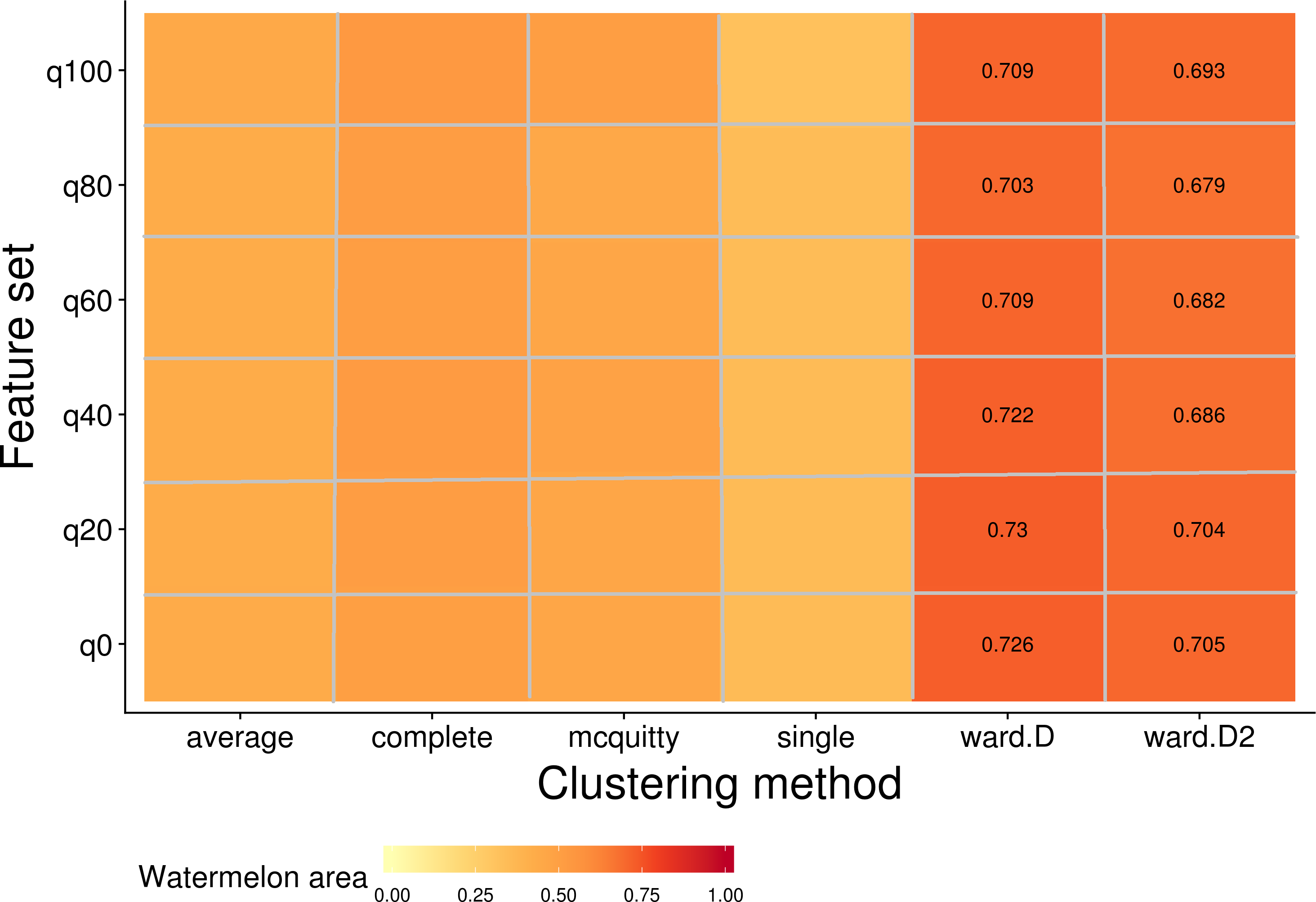


**Figure 9.** Heatmap of WM area depending on the remaining proportion of genes with higher read numbers (*y*-axis) and clustering methods (*x*-axis). The highest WM area values (starting from 75% of the maximum) are shown on the respective positions.


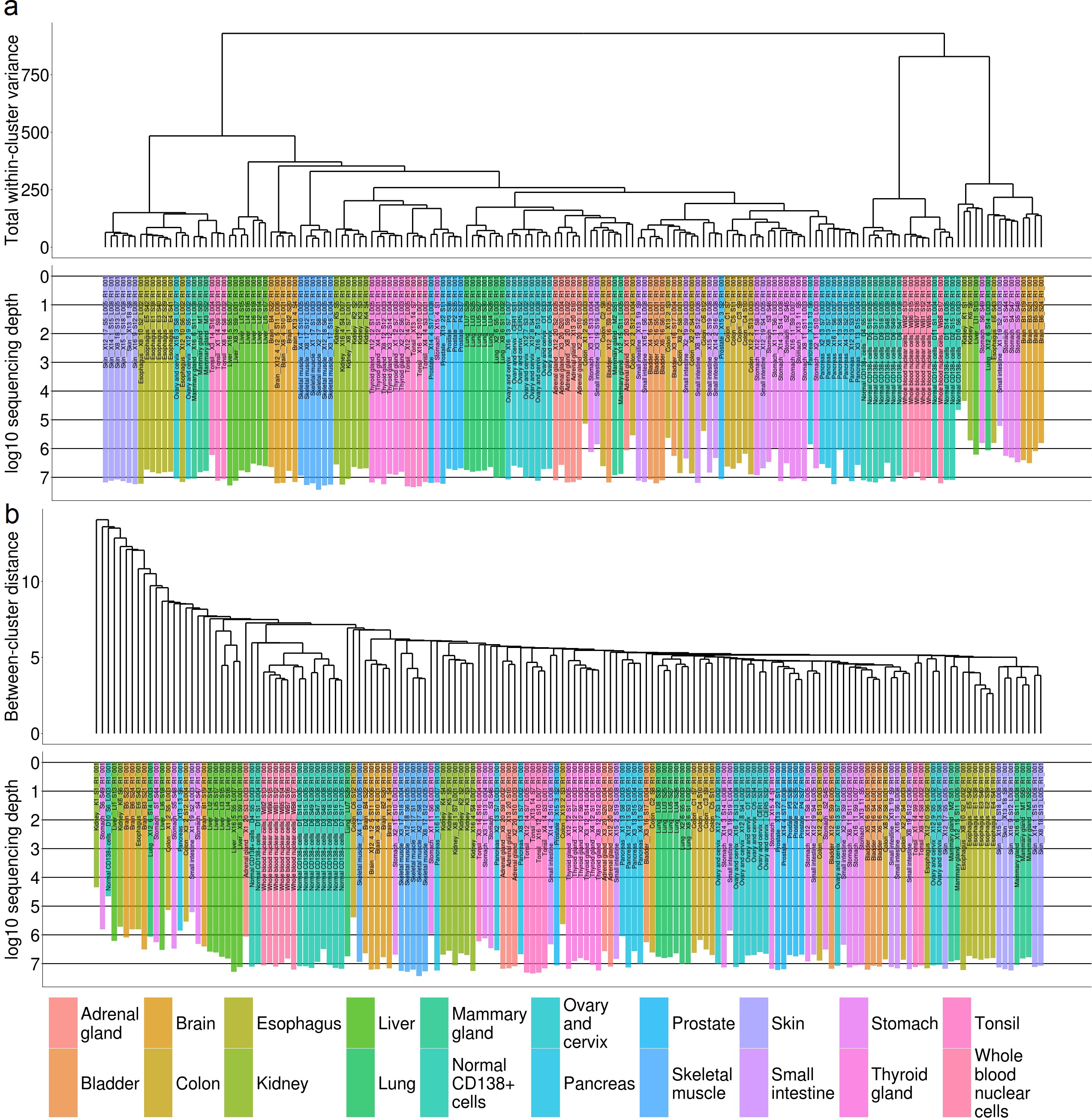


**Figure 10.** Dendrograms showing best (**a**) and worst (**b**) result of clustering normal human tissues. Tissue types are marked with colors and length of the bar is proportionate to log10 of total number of mapped raw counts for the respective RNA sequencing profile. Panel **a**: The best case. Q20 gene set and Ward.D algorithm settings were used. Panel **b**: The worst case. Q100 gene set and single linkage algorithm settings were used.

References.

1. Tomczak, K., Czerwinska, P. & Wiznerowicz, M. The Cancer Genome Atlas (TCGA): an immeasurable source of knowledge. *Contemporary Oncology (Poznan, Poland)* 19, A68–A77 (2015).

2. Love, M. I., Huber, W. & Anders, S. Moderated estimation of fold change and dispersion for RNA-seq data with DESeq2. *Genome Biology* 15, 550 (2014).

3. Forbes, S. A. *et al.* The Catalogue of Somatic Mutations in Cancer (COSMIC). in *Current Protocols in Human Genetics* (eds. Haines, J. L. et al.) (John Wiley & Sons, Inc., 2008).

4. Suntsova, M. *et al.* Atlas of RNA sequencing profiles for normal human tissues. *Scientific Data* 6, (2019).

5. Quinlan, J.R. Induction of decision trees. *Mach. Learn.* **1986**, *1*, 81–106.

6. Efron, B. & Tibshirani, R. On testing the significance of sets of genes. *The Annals of Applied Statistics* 1, 107–129 (2007).

7. Brock, G., Pihur, V., Datta, S. & Datta, S. clValid : An *R* Package for Cluster Validation. *Journal of Statistical Software* 25, (2008).

8. Handl, J., Knowles, J. & Kell, D. B. Computational cluster validation in post-genomic data analysis. *Bioinformatics (Oxford, England)* 21, 3201–3212 (2005).

9. Dunn, J. C. Well-Separated Clusters and Optimal Fuzzy Partitions. *Journal of Cybernetics* 4, 95–104 (1974).

10. Rousseeuw, P. J. Silhouettes: A graphical aid to the interpretation and validation of cluster analysis. *Journal of Computational and Applied Mathematics* 20, 53–65 (1987).

11. Zhu, Y., Qiu, P. & Ji, Y. TCGA-Assembler: open-source software for retrieving and processing TCGA data. *Nature Methods* 11, 599-600 (2014).

12. Clifford, H., Wessely, F., Pendurthi, S. & Emes, R. D. Comparison of clustering methods for investigation of genome-wide methylation array data. *Frontiers in Genetics* 2, 88 (2011).

1. * We call this score *watermelon area* since in some cases the plot looks like a watermelon slice, i. e. when the observed trajectory is very close to 95% of random data. [↑](#footnote-ref-1)
