# Supplementary material for: Disparity between Inter-Patient Molecular Heterogeneity and Repertoires of Target Drugs Used for Different Types of Cancer in Clinical Oncology
Source: Int J Mol Sci. 2020 Feb 26;21(5):1580. doi: 10.3390/ijms21051580 (PMC7084891; doi:10.3390/ijms21051580)
Supplement: Supplementary file 1 [file ijms-21-01580-s001.zip › ijms-691043-supplementary/Supplementary File 4AB.docx]

**Supplementary File 4. Correlation between cancer type-specific average pairwise distances for gene expression and mutation data, calculated separately for cancer stages I-IV.**


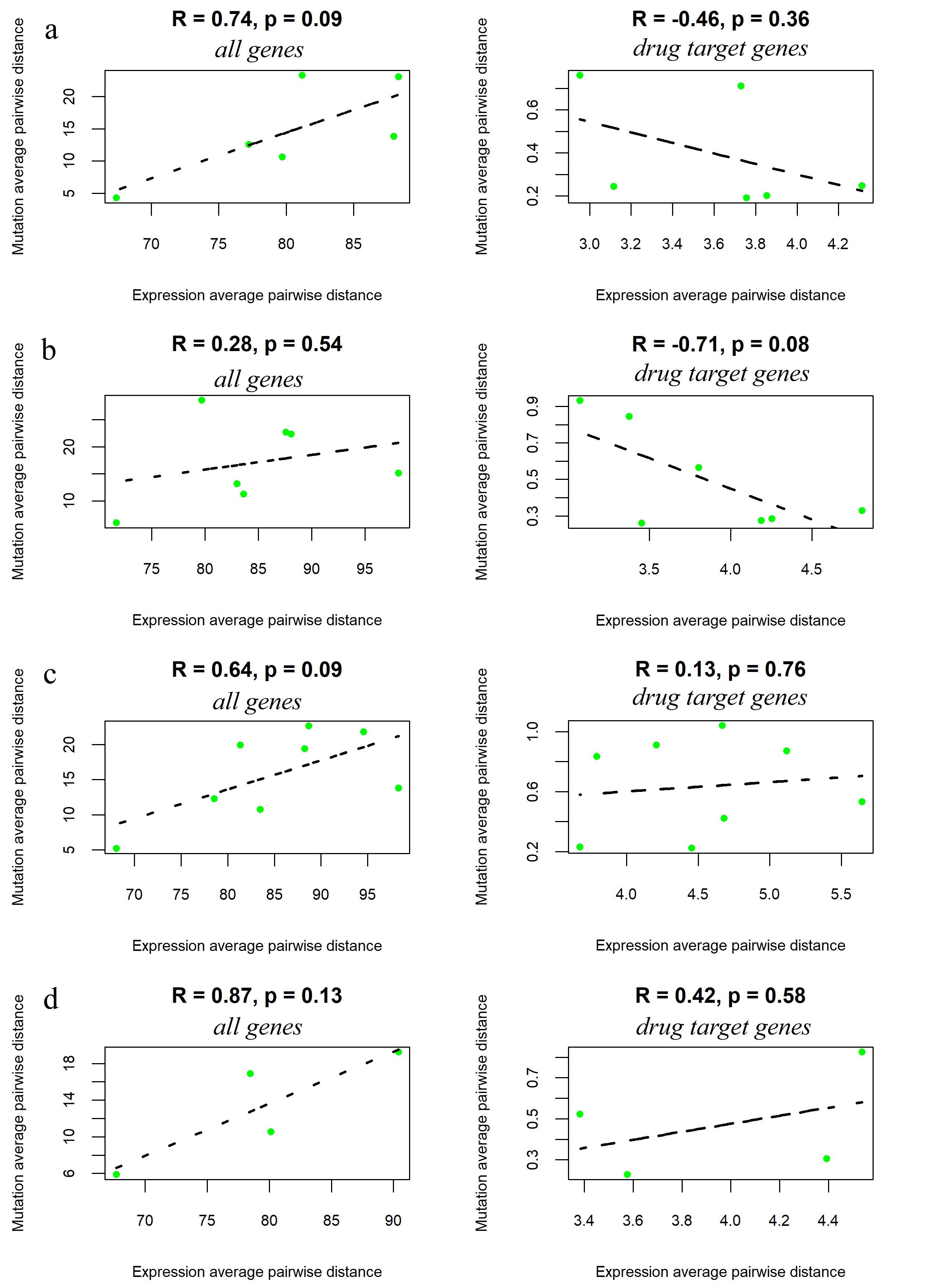


**Figure S1.** Correlation between average pairwise distances for gene expression and mutation data. Each dot corresponds to one cancer type, R is Pearson correlation coefficient. Correlations were calculated for molecular profiles of all genes (left) and for molecular profiles of drug target genes (right). **(a)** Correlation for stage I tumor samples. **(b)** Correlation for stage II tumor samples. **(c)** Correlation for stage III tumor samples **(d)** Correlation for stage IV tumor samples.
