# Supplementary material for: Disparity between Inter-Patient Molecular Heterogeneity and Repertoires of Target Drugs Used for Different Types of Cancer in Clinical Oncology
Source: Int J Mol Sci. 2020 Feb 26;21(5):1580. doi: 10.3390/ijms21051580 (PMC7084891; doi:10.3390/ijms21051580)
Supplement: Supplementary file 1 [file ijms-21-01580-s001.zip › ijms-691043-supplementary/Supplementary File 5AB.docx]

**Supplementary File 5. Clustering of cancer types under investigation by molecular profiles and targets of NCCN-recommended drugs, performed separately for cancer stages I-IV.**


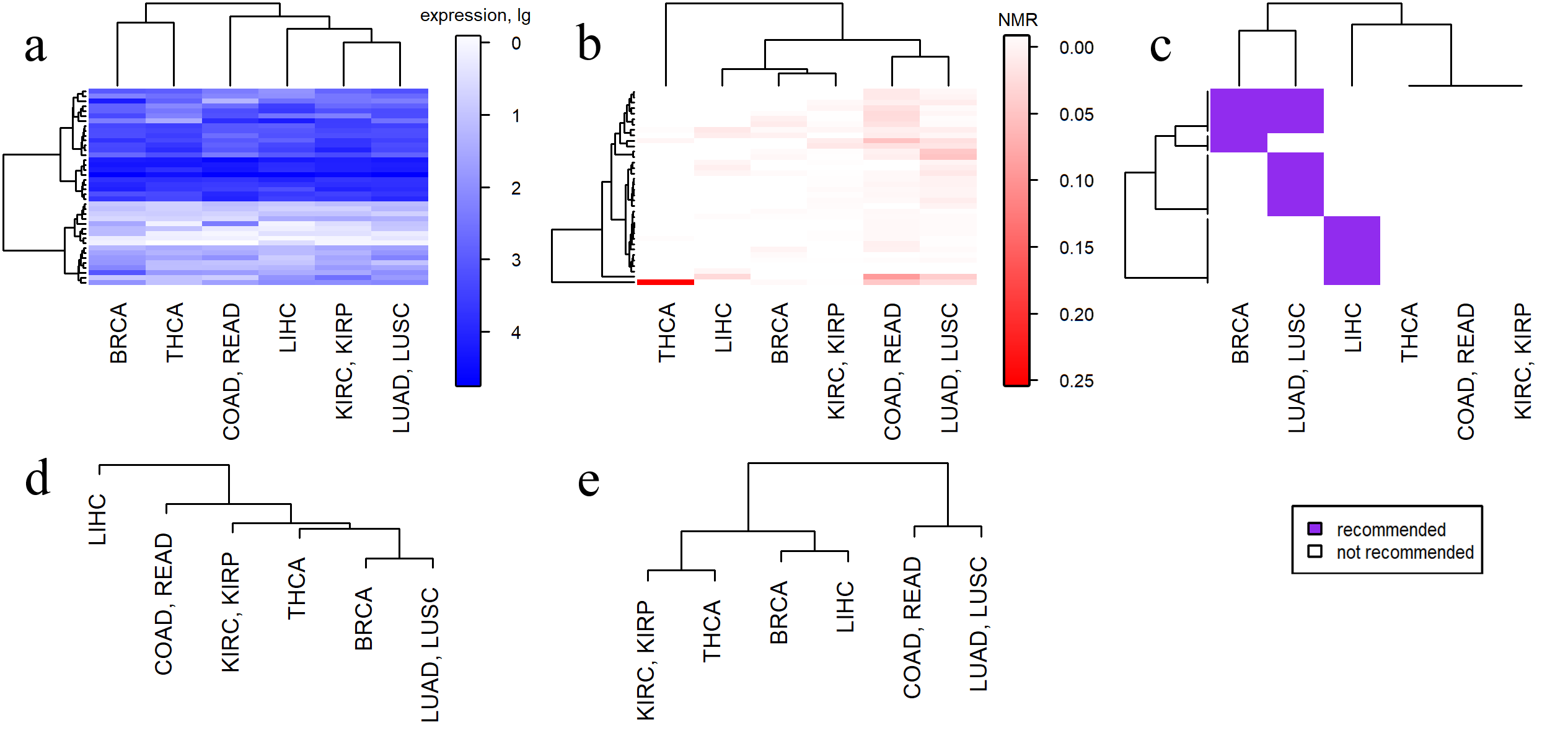


**Figure S1.** Clustering of cancer types where stage I profiles were available by molecular profiles and targets of NCCN-recommended drugs. Clustering by (a) averaged expression profiles (logarithm of Deseq2 normalized expression counts) for target genes of NCCN-recommended drugs for stage I cancers, (b) averaged mutation profiles (NMR) for target genes of NCCN-recommended drugs for stage I cancers, (c) molecular targets of NCCN-recommended drugs for stage I cancers, (d) expression profiles (logarithm of Deseq2 normalized expression counts) for all genes, (e) mutation profiles (NMR) for all genes. Cancer type abbreviations: BRCA - Breast invasive carcinoma, COAD - Colon adenocarcinoma, READ - Rectum adenocarcinoma, KIRC - Kidney renal clear cell carcinoma, KIRP - Kidney renal papillary cell carcinoma, LIHC - Liver Hepatocellular carcinoma, LUAD - Lung adenocarcinoma, LUSC - Lung squamous cell carcinoma, THCA - Thyroid carcinoma.


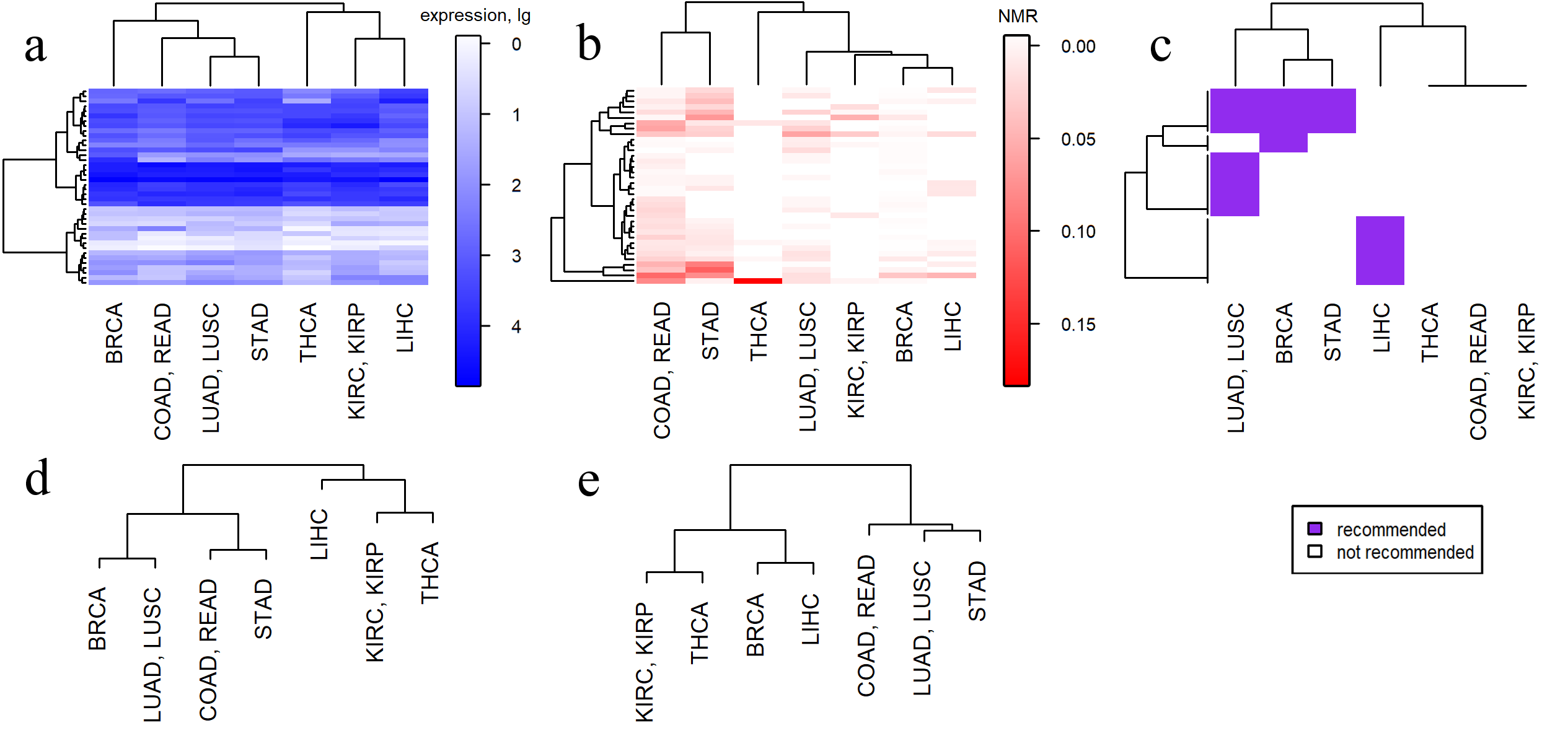


**Figure S2.** Clustering of cancer types where stage II profiles were available by molecular profiles and targets of NCCN-recommended drugs. Clustering by (a) averaged expression profiles (logarithm of Deseq2 normalized expression counts) for target genes of NCCN-recommended drugs for stage II cancers, (b) averaged mutation profiles (NMR) for target genes of NCCN-recommended drugs for stage II cancers, (c) molecular targets of NCCN-recommended drugs for stage II cancers, (d) expression profiles (logarithm of Deseq2 normalized expression counts) for all genes, (e) mutation profiles (NMR) for all genes. Cancer type abbreviations: BRCA - Breast invasive carcinoma, COAD - Colon adenocarcinoma, READ - Rectum adenocarcinoma, KIRC - Kidney renal clear cell carcinoma, KIRP - Kidney renal papillary cell carcinoma, LIHC - Liver Hepatocellular carcinoma, LUAD - Lung adenocarcinoma, LUSC - Lung squamous cell carcinoma, STAD - Stomach adenocarcinoma, THCA - Thyroid carcinoma.


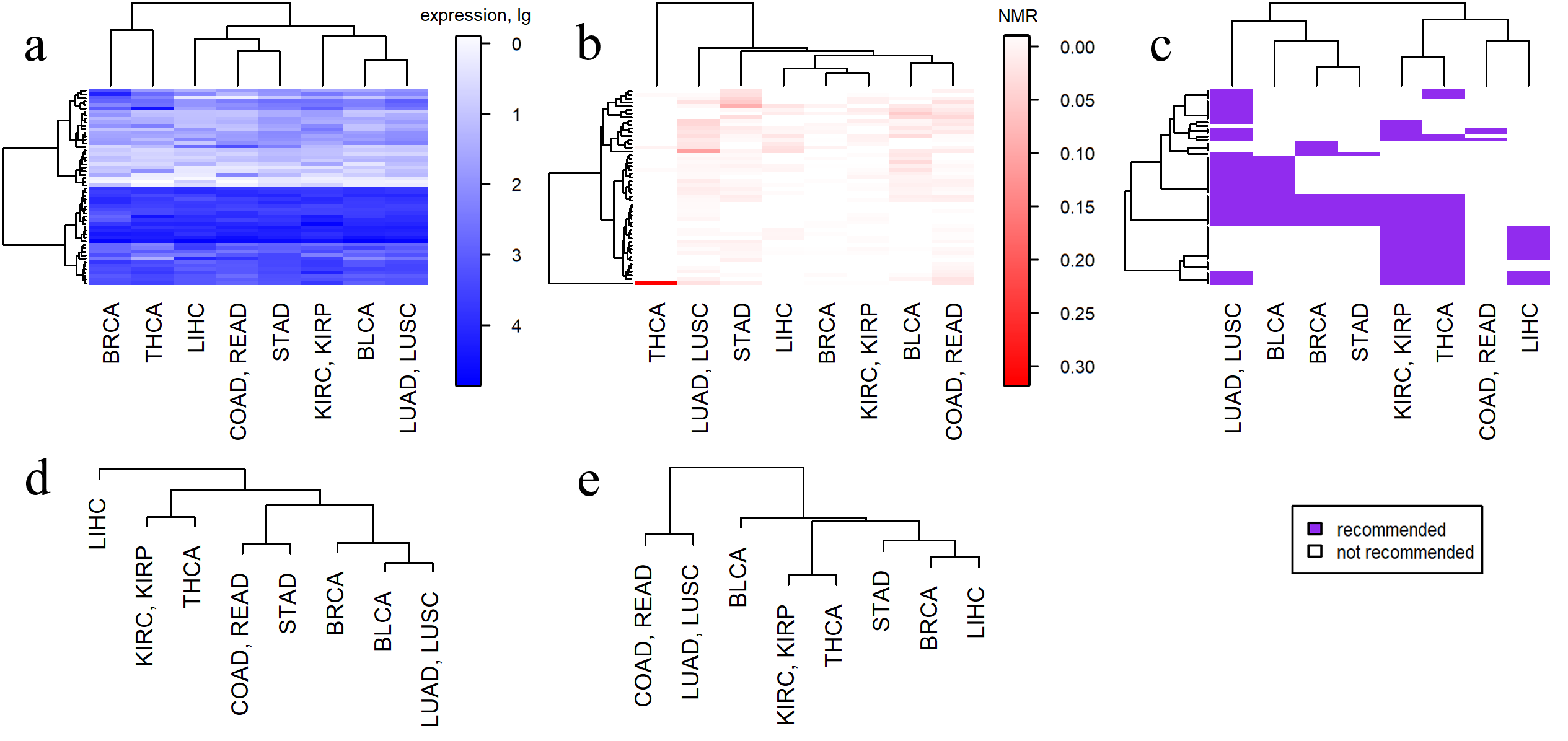


**Figure S3.** Clustering of cancer types where stage III profiles were available by molecular profiles and targets of NCCN-recommended drugs. Clustering by (a) averaged expression profiles (logarithm of Deseq2 normalized expression counts) for target genes of NCCN-recommended drugs for stage III cancers, (b) averaged mutation profiles (NMR) for target genes of NCCN-recommended drugs for stage III cancers, (c) molecular targets of NCCN-recommended drugs for stage III cancers, (d) expression profiles (logarithm of Deseq2 normalized expression counts) for all genes, (e) mutation profiles (NMR) for all genes. Cancer type abbreviations: BLCA -Bladder urothelial carcinoma, BRCA - Breast invasive carcinoma, COAD - Colon adenocarcinoma, READ - Rectum adenocarcinoma, KIRC - Kidney renal clear cell carcinoma, KIRP - Kidney renal papillary cell carcinoma, LIHC - Liver Hepatocellular carcinoma, LUAD - Lung adenocarcinoma, LUSC - Lung squamous cell carcinoma, STAD - Stomach adenocarcinoma, THCA - Thyroid carcinoma.


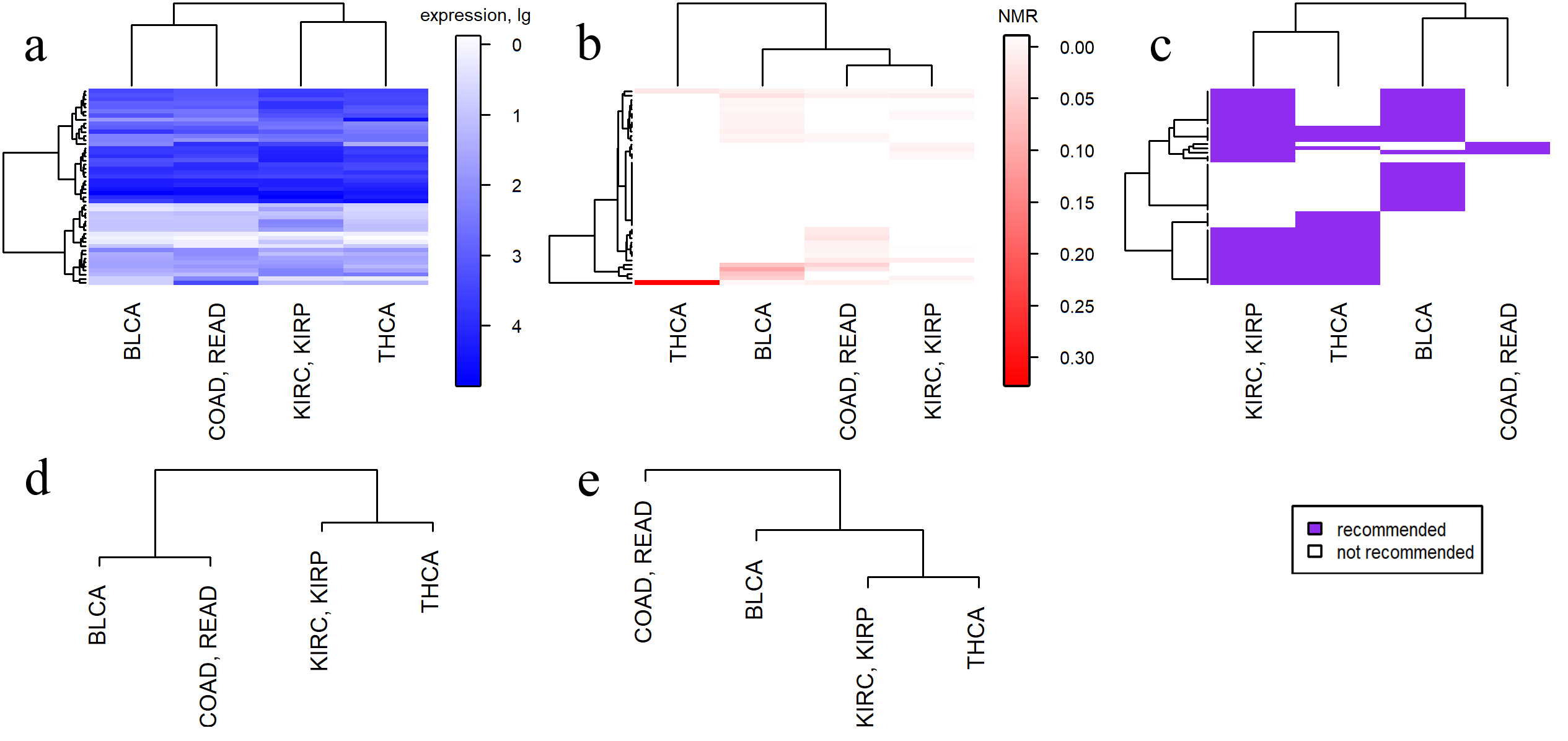


**Figure S4.** Clustering of cancer types where stage IV profiles were available by molecular profiles and targets of NCCN-recommended drugs. Clustering by (a) averaged expression profiles (logarithm of Deseq2 normalized expression counts) for target genes of NCCN-recommended drugs for stage IV cancers, (b) averaged mutation profiles (NMR) for target genes of NCCN-recommended drugs for stage IV cancers, (c) molecular targets of NCCN-recommended drugs for stage IV cancers, (d) expression profiles (logarithm of Deseq2 normalized expression counts) for all genes, (e) mutation profiles (NMR) for all genes. Cancer type abbreviations: BLCA -Bladder urothelial carcinoma, COAD - Colon adenocarcinoma, READ - Rectum adenocarcinoma, KIRC - Kidney renal clear cell carcinoma, KIRP - Kidney renal papillary cell carcinoma, THCA - Thyroid carcinoma.­­­
